# Socioeconomic equity in maternal health services use in Bangladesh: The role of service readiness in health facilities during the period 2001–2016

**DOI:** 10.1371/journal.pone.0354897

**Published:** 2026-07-30

**Authors:** Karar Zunaid Ahsan, Gustavo Angeles, Siân L. Curtis, Peter Kim Streatfield, Kavita Singh

**Affiliations:** 1 Department of Public Health Leadership & Practice, Gillings School of Global Public Health, the University of North Carolina at Chapel Hill, North Carolina, United States of America; 2 Department of Maternal and Child Health, Gillings School of Global Public Health, the University of North Carolina at Chapel Hill, North Carolina, United States of America; 3 Health System and Population Studies Division (HSPSD), International Centre for Diarrhoeal Disease Research, Bangladesh (icddr, b), Dhaka, Bangladesh; International Centre for Diarrhoeal Disease Research, Bangladesh (icddr, b), BANGLADESH

## Abstract

**Background:**

Equity in health service utilization refers to equal treatment for equal health needs, regardless of socioeconomic status. In low- and middle-income countries, inequity in maternal health service use remains a pressing issue in the pursuit of universal health coverage by 2030. This study examines whether socioeconomic equity in the use of key maternal health services—antenatal care (ANC) from a medically trained provider, facility delivery, and management of complications—improved faster in districts with high facility readiness than elsewhere in Bangladesh between 2001 and 2016.

**Methods and findings:**

We analyzed data from three rounds of the Bangladesh Maternal Mortality and Health Care Survey and corresponding Bangladesh Health Facility Survey rounds. These datasets were linked at the district level and pooled for the years 2001, 2010, and 2016. Using an adaptation of the Difference-in-Differences (DID) model based on linear probability models, we estimated whether poor-nonpoor gaps in maternal health service utilization changed differently over time in districts with high facility readiness than in districts with lower readiness. Bangladesh achieved substantial improvements in overall maternal health service use during the study period. However, these gains were not equitably distributed. Socioeconomic equity improved modestly for ANC and complication management but worsened significantly for facility deliveries. The gap between nonpoor and poor women widened more in high facility readiness districts than in low readiness districts (p < 0.05). Additionally, equity in ANC use improved among women living within one hour of a private facility (p < 0.05).

**Conclusions:**

This is the first systematic investigation of the role of facility readiness in maternal health equity in Bangladesh using nationally representative data. Despite overall progress, persistent and, in some cases, widening socioeconomic inequities were observed—especially in facility-based delivery. These findings highlight the need for targeted policy action to ensure that improvements in service readiness translate into more equitable maternal healthcare access and outcomes.

## Background

Equity in health services involves providing equal treatment for equal health needs, regardless of socioeconomic status [1]. While differences in health status are partly linked to biological factors, health disparities negatively impact groups facing systematic social or economic obstacles, discrimination, or exclusion [2]. Addressing these inequities is morally imperative and economically beneficial by reducing treatment costs and productivity losses [3]. In particular, maternal health often reflects the status of women and the effectiveness of healthcare systems in a country [4]. Improving maternal health through high-quality antenatal care, facility-based delivery, postnatal care, and complication management is central to global development priorities, initially included in the Millennium Development Goals (MDGs) and now integral to achieving universal health coverage under the Sustainable Development Goals (SDGs) [5–7]. Almost all pregnancy-related deaths occur in low- and middle-income countries and are preventable with timely, quality maternal healthcare. Despite significant reductions in maternal mortality during the MDGs era (2000–2015), progress remained inequitable. Persistent disparities by economic, educational, and geographic factors continue to challenge equitable maternal healthcare utilization, a critical priority for achieving the SDGs [8,9].

Following a 40% reduction in the maternal mortality ratio (MMR) between 2001 and 2010, Bangladesh was considered one of only nine countries on track to achieve the MDG 5 target by 2015 [10]. However, a recent study found that the decline in MMR stagnated between 2010 and 2015 despite increases in maternal health services use (viz., ANC, facility delivery, PNC, medical care-seeking for management of pregnancy complications) as well as an overall socioeconomic improvement [11]. Since improving equity in maternal health service use can improve maternal health and reduce maternal mortality [12], the Government of Bangladesh has focused on expanding the provision of maternal health services at health facilities through national health sector programs. Since 1998, Bangladesh has implemented four consecutive health sector programs and made substantial progress in health systems strengthening that improved functionality of health facilities to provide essential care [13]. Bangladesh, therefore, makes an interesting case study to examine how the socioeconomic inequity in maternal health services use has evolved during 2001‒2016 and investigate the role of health facility readiness in this evolution.

**Fig 1:**
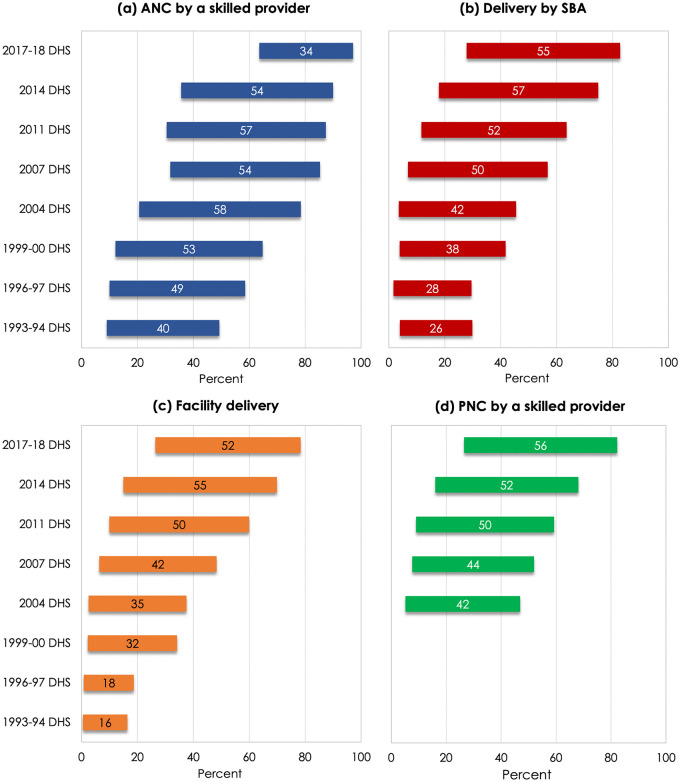
Trends in poorest-richest inequity in the use of maternal health services, Bangladesh 1993–2018. ^a^ Inequity is defined as the percentage point gap between the poorest and the richest wealth quintiles. ^b^ Data from Bangladesh Demographic and Health Surveys, multiple rounds.

**Fig 2:**
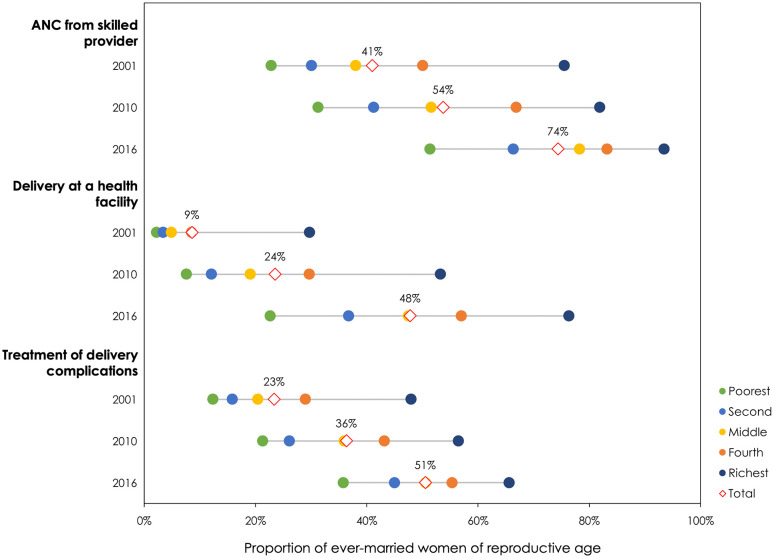
Trends in maternal health services use by socioeconomic status, Bangladesh 2001‒2016^a^. **^a^** Fig 2 is an equiplot: each dot represents the level of service use within a wealth quintile, and the horizontal distance between the dots represents the size of the socioeconomic gap.

Overall utilization of critical reproductive health services also increased steadily during 2004‒2018. ANC from a medically-trained provider increased from 20% to 82%, the proportion of births delivered by a skilled birth attendant (SBA) increased from 9% to 53%, facility delivery increased from 4% to 50%, and PNC increased from 18% to 53% (2004‒2018) [14]. However, the gap in maternal health service use between the poorest and richest wealth quintiles has widened over time (see Fig 1). For ANC, the gap narrowed only after 2014, while for SBA and facility delivery, it substantially increased between 1993 and 2018. For PNC, available data (2004–2018) indicate a steady widening of the gap [14]. As defined in its caption, Fig 1 presents the summary percentage-point gap between the poorest and richest wealth quintiles rather than wealth-quintile–specific levels; the underlying utilization patterns by individual wealth quintile are presented in Fig 2.

Inequity is observed outside the health sector as well. For example, the Human Development Index (HDI) reflects Bangladesh’s socioeconomic development. Between 1990 and 2019, the HDI value for Bangladesh has improved by 60%. It currently is at 0.632—which is in the medium human development category—positioning the country at 133 out of 189 countries and territories [15]. However, if we adjust for inequality (see technical notes on calculating the inequality-adjusted HDIs for methodological details) [16], Bangladesh’s HDI falls to 0.478 for 2019—a loss of 24% due to the disparity in the distribution of the HDI dimensions (i.e., life expectancy, education, and per capita income) [15]. While the persistent socioeconomic disparity in Bangladesh could be a possible explanation for the observed gap in MHC services use between the poorest and the richest quintiles, there is no systematic assessment of health system determinants of MHC services use. Available studies focused on either the levels and trends of inequity in maternal health in Bangladesh [17–25] or the impact of specific health programs on inequity in MHC services use based on small geographic regions [26–30]. Studies on levels and trends in maternal health inequity were analyzed from household sample surveys, which only allowed examination of ‘demand-side’ factors of MHC services use. These studies found that socioeconomic (viz., poor-nonpoor) and geographic (viz., urban-rural) disparities continued to exist over the years [17–21], and women’s education, religion, wealth index, and region are important demand-side factors in seeking MHC services in Bangladesh [20].

In this paper, we primarily aimed to understand the role of supply-side factors, such as facility readiness, in maternal health services use to fill a crucial knowledge gap. For this study, we hypothesize that gradual improvement in overall socioeconomic status and the government’s health systems strengthening initiatives were associated with changes in equity in maternal healthcare use during the study period. Against this background, the objective of our study was to examine the role of socioeconomic status and select supply-side factors (viz., the readiness of health facilities and availability of private sector facilities) during this period, after controlling for relevant socio-demographic factors of maternal health services use. More specifically, we would like to assess whether the socioeconomic inequity decreased more in high facility readiness districts than in the rest of the country.

## Methods

### Data sources

We analyzed demand-side determinants of maternal health services (ANC from a medically-trained provider, facility delivery, and complication management) using data from three rounds (2001, 2010, 2016) of the nationally representative Bangladesh Maternal Mortality and Health Care Survey (BMMS). The BMMS covered large household samples (approximately 100,000 in 2001, 174,000 in 2010, and 298,000 in 2016) and provided comparable data on maternal health indicators, socioeconomic characteristics, and healthcare utilization among ever-married women aged 13–49 who had a live birth in the preceding three years [31–33]. To ensure that the data are nationally representative, BMMS survey weights are not calculated only at the Primary Sampling Unit (PSU) level; they are calculated using a two-stage sample design that accounts for probabilities at both the PSU (first stage) and household (second stage) levels within specific strata. The final household survey datasets were accessed on November 16, 2021, and the authors did not have access to information that could identify individual participants during or after data collection.

Supply-side determinants were assessed using three rounds (2000, 2011, 2017) of the Bangladesh Health Facility Survey (BHFS)/Service Provision Assessment (SPA). These nationally representative surveys employed stratified random sampling (855 facilities in 2000, 1,503 in 2011, and 1,524 in 2017) to assess readiness and provision of essential health services by public, NGO, and private facilities, specifically those registered with the Bangladesh government’s Ministry of Health. BHFS/SPA survey weights are not calculated solely at the PSU level; they are derived using a multi-stage approach that incorporates PSU-level selection probabilities, facility-level sampling, and adjustments for non-response at both the facility and provider/client levels to ensure national representativeness. Details of the sampling methodology and data collection can be found in the BHFS/SPA final reports [34–36].

This study used publicly available secondary, de-identified data from nationally representative household and health facility surveys by the National Institute of Population Research and Training (NIPORT), the DHS Program, and the MEASURE Evaluation project. The surveys were approved by the respective organization’s Institutional Review Board and the National Research Ethics Committee of the Bangladesh Medical Research Council. Informed verbal consent was obtained from survey participants by the original study teams; for the present secondary analysis of anonymized data, additional review was deemed exempt by the Office of Human Research Ethics at the University of North Carolina at Chapel Hill under the regulatory category of Human Subject Research, in accordance with 45 CFR 46.104.

### Variables

As outcomes, this study focuses on three dichotomous variables of maternal health services—ANC visits from a medically trained provider during pregnancy, delivery in a health facility, and medical care-seeking for management of pregnancy and/or delivery complications. A summary of the variables used in this analysis is provided in Table 1.

**Table 1:** Summary of variables for analysis.

Classification	Domain	Variables	Definition	Coding
Outcome	Maternal health services	Antenatal care	Woman sought at least one ANC from a medically trained provider	Dichotomous
		Facility delivery	Woman delivered in a health facility	Dichotomous
		Management of complications	Woman with a complication during pregnancy/delivery sought care from a trained provider/health facility	Dichotomous
Demand-side predictors	Socioeconomic	Household wealth	Socioeconomic status based on household assets and amenities	Categorical/ Dichotomous
		Maternal education	Completed years of schooling	Categorical
		Rurality	Current place of residence	Dichotomous
	Demographic	Mother’s age	Age in years at birth of the index child	Categorical
		Parity	Birth order of the index child	Categorical
Supply-side predictors	Structural	Facility readiness	District average of facility readiness for maternal health services	Categorical/ Dichotomous
		Access to health facility	Closest health facility (public and private separately) that provides maternal health services is within 1 hour of travel	Dichotomous

For this analysis, socioeconomic status was measured using a wealth index based on household’s ownership of selected durable assets and dwelling characteristics (e.g., assets like a television or a bicycle, and amenities such as the source of drinking water, toilet facilities, etc.), derived through principal component analysis [37]. Respondents were grouped into five wealth quintiles based on their household’s wealth index [14]. For the multivariate regression analysis to estimate how the poor–nonpoor gap in maternal health services use differed by facility readiness, we categorized poor (comprising the two lowest wealth quintiles) and nonpoor (comprising the remaining three higher wealth quintiles), consistent with definitions used for lower-middle-income countries like Bangladesh [38].

Facility readiness for delivery care was assessed using a composite score based on infrastructure, equipment, supplies, and commodities. Indicators were selected based on BHFS/SPA data and guidance from the World Health Organization (WHO)’s Service Availability and Readiness Assessment (SARA) manual, the Newborn Indicator Technical Working Group, and a recent systematic review [39–41] (see S1 Table in the supporting information). Each indicator was equally weighted, and scores were standardized to range from 0 to 1, representing the percentage of maximum possible readiness [42]. Given this standardization, a facility’s score is interpreted as the percentage of the highest possible readiness that the facility could have. This composite captures structural readiness (i.e., the physical infrastructure, equipment, supplies, and commodities required to provide maternal health services) and therefore measures a facility’s structural capacity to deliver care. It does not directly measure process quality, clinical quality, or patient experience, and we interpret it accordingly throughout this paper.

As the BHFS/SPA and BMMS surveys were independently sampled, we linked facility readiness to households at the district level—the lowest common geographic unit. District-level average readiness scores (the means of the district averages of facility readiness were 0.544 in 2000, 0.340 in 2011, and 0.322 in 2017) were merged into the pooled BMMS dataset (see S2 Table in the supporting information). Each respondent was assigned their district’s average score, and the distribution was divided into terciles (low, medium, high) to examine maternal health service use. For multivariate analysis, we grouped facility readiness into two categories: low (bottom two terciles) and high (top tercile) to assess the differential effects of readiness and socioeconomic status. District readiness classification was time-varying across survey rounds. For each BMMS-BHFS linked year, district-average readiness was calculated from the corresponding facility survey and districts were then classified relative to the distribution in that survey year; therefore, a district could move between readiness categories across waves. Because the household and facility surveys were sampled independently, households were not linked to individual facilities or to the nearest facility; instead, each district containing BMMS respondents was matched to the corresponding district-average readiness score from the same-round BHFS/SPA, so the linkage represents the broader district service environment rather than the specific facility a woman used. All districts with BMMS respondents were successfully linked to a readiness score. The separate proximity measures (nearest public or private facility within one hour) are distinct from this readiness linkage and are based on respondents’ self-reported travel time to the nearest facility.

### Statistical analysis

Socioeconomic disparities in maternal health services use in Bangladesh over time were measured by concentration indices, where the index value is bounded between ‒1 to +1, with zero indicating perfect equality. A positive concentration index indicates that service use is concentrated among wealthier (nonpoor) women; a larger positive value reflects greater pro-rich inequality, a decline in the index over time indicates a reduction in pro-rich inequality, and an increase indicates widening pro-rich inequality. As the outcomes were dichotomous, we applied Erreygers’ transformation using Stata’s “conindex” command to correct index limitations, ensuring indices remained within the [–1, 1] interval and satisfied essential analytical requirements [43].

To examine how the poor–nonpoor gap in service use changed over time across districts with differing facility readiness during 2001‒2016, we applied an adaptation of the Difference-in-Differences (DID) model. For this, we used linear probability model (LPM) on a pooled dataset of 2001, 2010, and 2016 BMMSs linked to corresponding BHFS/SPA with the following regression specification:


$$\begin{array}{c} {\;Y}_{ijt}=\beta_{\text{0}}+\beta_{\text{1}}S_{it}+\beta_{\text{2}}T_{t}+\beta_{\text{3}}S_{it}\cdot T_{t}+\beta_{\text{4}}Z_{jt} \end{array}$$

$$\begin{array}{c} \;+\beta_{\text{5}}Z_{jt}\cdot S_{it}+\beta_{\text{6}}Z_{jt}\cdot T_{t}+\beta_{\text{7}}Z_{jt}\cdot S_{it}\cdot T_{t}+\beta_{\text{8}}X_{ijt}+\varepsilon_{ijt} \end{array}$$ (1)

where,$Y_{ijt}$ is the outcome of interest for individual *i* who lives in district *j* at time *t*. $S_{it}$ takes the value of 1 if the woman *i* belongs *t*o nonpoor socioeconomic status (i.e., household wealth quintiles 3‒5) and 0 if she belongs to poor socioeconomic status (i.e., household wealth quintiles 1 and 2). $T_{t}$ is a vector of time dummies to represent the BMMS survey rounds 2010 and 2016. Z is an indicator variable that takes the value of 1 if the woman *i* lives in a district with high facility readiness (i.e., facility readiness tercile 3) and 0 if the individual lives in a district with low facility readiness for maternal health services (i.e., readiness terciles 1 and 2). $X_{ijt}$ are the relevant socio-demographic covariates influencing the outcomes.

In equation (1), $\beta_{\text{3}}$ measures the change in socioeconomic equity that occurred during the study period with low facility readiness, and $\left(\beta_{\text{3}}+\beta_{\text{7}}\right)$ measures the change in socioeconomic equity in districts with high facility readiness (coefficients were combined using “lincom” command in Stata). Finally, $\beta_{\text{7}}$ indicates the differential change in equity between high- and low-readiness districts, after controlling for relevant covariates, during the study period (see S3 Text in the supporting information for a detailed description of how the LPM coefficients were identified to measure equity). This specification is not intended to evaluate a single policy intervention with a discrete adoption date. Rather, it compares whether poor–nonpoor differences in service use changed differently over time across district-year service environments defined by facility readiness. Accordingly, the triple interaction term should be interpreted as a difference in the change in the poor–nonpoor gap between higher- and lower-readiness districts over time, conditional on observed covariates. In plain terms, the difference-in-differences (DID) estimate captures how much the poor–nonpoor gap in service use changed over time, while the difference-in-difference-in-differences (DIDID) estimate captures whether that change in the gap was larger or smaller in high-readiness districts than in low-readiness districts.

All the analyses were performed using STATA v.16 [44], and observations with missing data on model variables were excluded from regression analyses (complete-case analysis). Missing data on the model variables were limited, with 1.12% of otherwise-eligible observations excluded from the regression models; the extent of missingness did not differ substantially across survey rounds or readiness groups. We used appropriate survey sampling weights, tested for multicollinearity, and controlled for clustering and heteroscedasticity to obtain robust standard errors. Standard errors were clustered at the cluster level. The specification includes survey-round (time) indicators and a district-level readiness indicator but does not include district fixed effects; it is therefore a pooled repeated cross-sectional interaction model rather than a two-way fixed-effects difference-in-differences design, which is what we mean by an “adaptation” of the DID approach. We chose LPM over logistic and probit regression for binary outcomes in this study due to its interpretability and widespread use in economics and social sciences [45]. Additionally, when the binary outcome is common (i.e., prevalence >10%), logistic regression’s odds ratio can misrepresent the true association and LPM is effective for analyzing binary outcomes in such cases [46].

## Results

### Respondent’s characteristics

Between 2001 and 2016, there were notable increases in urbanization, women’s educational attainment, and availability of health facilities in Bangladesh (see Table 2). In particular, there was a massive growth in the private health sector during this period—only 17% of recent mothers lived within one hour of travel from a private health facility in 2001, which increased to 84% in 2016. Fertility also reduced during this period, as seen in the shift towards lower parity among the respondents.

**Table 2:** Distribution (%) of demographic, socioeconomic, and structural characteristics of respondents, Bangladesh 2001‒2016.

Background characteristics	Year		
	2001	2010	2016
Woman’s age at childbirth			
<18	10.0	5.1	12.2
18-24	43.3	48.6	49.0
25-29	22.8	26.2	23.9
30-34	14.3	12.8	11.2
35-39	6.8	5.4	3.1
40-49	2.8	1.9	0.7
Parity			
1	26.5	32.1	38.5
2‒3	43.2	47.8	49.7
4+	30.3	20.1	11.9
Locality			
Urban	17.3	23.0	26.5
Rural	82.7	77.0	73.5
Women’s education			
No schooling	46.2	23.9	9.6
Any primary	29.2	31.5	29.5
Secondary incomplete	17.8	34.5	40.8
Secondary complete or higher	6.7	10.2	20.2
Household’s socioeconomic status			
Poorest	25.0	22.4	20.1
Second	21.9	19.7	20.3
Middle	19.0	20.1	19.7
Fourth	17.6	18.9	20.6
Richest	16.5	18.8	19.3
Time to reach the nearest public health facility			
>1 hour	29.5	11.7	10.0
<1 hour	70.6	88.3	90.1
Time to reach the nearest private health facility			
>1 hour	82.7	31.9	16.2
<1 hour	17.3	68.1	83.8
Facility readiness for maternal health services (district-level)			
Low	36.8	32.6	31.7
Medium	31.3	34.8	31.8
High	31.9	32.6	36.5
*Observations*	*39,525*	*18,256*	*27,188*

^a^Respondents are ever-married women who gave birth in the last three years.

### Trends in maternal health services use during 2001‒2016

Overall, use of medically trained antenatal care increased from 41% (2001) to 74% (2016), while facility deliveries rose from 9% to 48%. Around half of the women reported pregnancy or delivery complications, with medically trained treatment increasing from 23% to 51%. Despite significant improvements (p < 0.05) in all services, utilization gains were uneven across socioeconomic groups.

Fig 2 illustrates maternal health service use by wealth quintiles over time, highlighting absolute inequities [47]. It shows a modest narrowing in the rich-poor gap for ANC and complication management but a significant widening for facility deliveries. In 2016, service use among the richest quintile was substantially higher compared to the poorest.

### Changes in equity in service use by socioeconomic status

By examining the concentration indices for 2001, 2010, and 2016, we can see that inequity in maternal health services use continued to favor the better off in Bangladesh during the study period—i.e., services were used disproportionately more by women belonging to higher wealth quintiles than their counterparts (see Table 3). Because the concentration index is a summary measure that aggregates inequality across the full wealth distribution, it does not show how use changed within individual wealth quintiles; the corresponding wealth-quintile–specific patterns are presented in Fig 2. There were clear indications that inequities in ANC and treatment for delivery/pregnancy complications have reduced significantly (p < 0.001) over the years. However, the concentration index values increased by 135% between 2001 and 2016 for delivery at a health facility, which indicated a statistically significant (p < 0.001) increase in socioeconomic inequity in service use.

**Table 3:** Concentration indices for maternal health services use by socioeconomic status, Bangladesh 2001‒2016.

Year	Concentration Index (CI)	95% Confidence Interval	% change in CI			F-statistic
			*‘01*‒*’10*	*‘10*‒*’16*	*‘01*‒*’16*	
ANC from a medically trained provider						
2001	0.385	0.354‒0.416	6%	‒21%	‒16%	26.68^**^
2010	0.406	0.383‒0.430				
2016	0.322	0.297‒0.347				
Delivery at a health facility						
2001	0.173	0.138‒0.207	99%	18%	135%	843.46^**^
2010	0.343	0.322‒0.364				
2016	0.405	0.383‒0.428				
Treatment of delivery complications						
2001	0.253	0.225‒0.281	11%	‒21%	‒12%	7.45^**^
2010	0.280	0.251‒0.309				
2016	0.222	0.195‒0.250				

**Note 1:** F-statistic from the test for statistically significant differences with H_0_: Difference of CI between years = 0.

**Note 2:** ^**^ p < 0.001; ^*^ p < 0.05; ^†^ p < 0.10 denote significance level of the F-statistic.

### Differential changes in equity by facility readiness in maternal health services use

**Fig 3:**
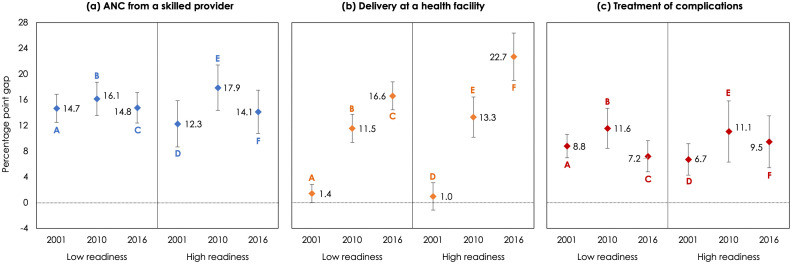
LPM coefficients showing changes in inequity in health services use by facility readiness^a,b^. ^**a**^ LPM coefficients from Table 4 corresponding to the data points are as follows: A = Nonpoor; B = Nonpoor + Nonpoor×2010; C = Nonpoor + Nonpoor×2016; D = Nonpoor + Nonpoor×High; E = Nonpoor + Nonpoor×2010 + Nonpoor×High + Nonpoor×High×2010; and F = Nonpoor + Nonpoor×2016 + Nonpoor×High + Nonpoor×High×2016. ^b^ Error bars represent 95% confidence intervals.

Fig 3 shows the socioeconomic inequity, measured as the percentage point gap in maternal health services use among the nonpoor and the poor, by facility readiness during the study period. Between 2001 and 2016, socioeconomic inequity for ANC remained similar in low-readiness districts (see points A‒C in Fig 3-a) and increased by 1.9 percentage points in high-readiness districts (see points D‒F in Fig 3-a). The DID model estimated that the gap between poor and nonpoor increased by 1.8 percentage points between low and high facility readiness areas during this period, which is the difference-in-difference-in-difference (DIDID) estimate (see the coefficient of ‘Nonpoor×High×2016’ under the column for ANC in Table 4). Since the DIDID estimate was not statistically significant, we conclude that the differential change in the poor–nonpoor gap for ANC across readiness groups was negligible between 2001 and 2016.

**Table 4:** Differential changes in socioeconomic equity by facility readiness, Bangladesh 2001‒2016.

Background Characteristics	Skilled ANC	Facility delivery	Complication treatment
Woman’s age at childbirth (reference category: < 18 years)			
18-24	0.038^**^	0.035^**^	0.054^**^
25-29	0.085^**^	0.091^**^	0.108^**^
30-34	0.112^**^	0.126^**^	0.141^**^
35-39	0.088^**^	0.134^**^	0.159^**^
40-49	0.078^**^	0.135^**^	0.166^**^
Parity (reference category: 1)			
2-3	−0.073^**^	−0.098^**^	−0.063^**^
4 or more	−0.161^**^	−0.170^**^	−0.101^**^
Place of residence (reference category: urban)			
Rural	−0.076^**^	−0.081^**^	−0.059^**^
Women’s education (reference category: no schooling)			
Any primary	0.096^**^	0.016^**^	0.060^**^
Secondary incomplete	0.230^**^	0.114^**^	0.163^**^
Secondary complete or higher	0.346^**^	0.318^**^	0.280^**^
Time to reach public health facility (reference category: > 1 hour)			
<1 hour	0.060^**^	0.024^**^	0.038^**^
Time to reach private health facility (reference category: > 1 hour)			
<1 hour	0.026^*^	0.035^**^	0.022^*^
Facility readiness (reference category: low readiness)			
High readiness	−0.016	−0.004	−0.003
Socioeconomic status (reference category: poor)			
Nonpoor	0.147^**^	0.014^*^	0.088^**^
Survey round (reference category: 2001)			
2010	0.011	0.009	0.035^*^
2016	0.170^**^	0.185^**^	0.174^**^
Interaction between socioeconomic status and survey round			
Nonpoor×2010	0.015	0.101^**^	0.028
Nonpoor×2016	0.001	0.152^**^	−0.016
Interaction between facility readiness and survey round			
High readiness×2010	−0.003	0.020	−0.001
High readiness×2016	0.004	−0.056^*^	−0.048^*^
Interaction between socioeconomic status and facility readiness			
Nonpoor×High readiness	−0.024	−0.004	−0.021
Interaction of socioeconomic status, facility readiness, and survey round			
Nonpoor×High x 2010	0.041	0.022	0.016
Nonpoor×High x 2016	0.018	0.065^*^	0.043
Constant	0.281^**^	0.105^**^	0.117^**^
Nonpoor×2010 + Nonpoor×High×2010	0.056^*^	0.123^**^	0.043
Nonpoor×2016 + Nonpoor×High×2016	0.019	0.217^**^	0.027
Nonpoor + Nonpoor×2010	0.161^**^	0.115^**^	0.116^**^
Nonpoor + Nonpoor×2016	0.148^**^	0.166^**^	0.072^**^
Nonpoor + Nonpoor×High	0.123^**^	0.010	0.067^**^
Nonpoor+Nonpoor×2010 + Nonpoor×High+Nonpoor×High×2010	0.179^**^	0.133^**^	0.112^**^
Nonpoor+Nonpoor×2016 + Nonpoor×High+Nonpoor×High×2016	0.141^**^	0.227^**^	0.095^**^

** p < 0.001; * p < 0.05; † p < 0.10.

For facility delivery, socioeconomic inequity increased by 15.2 percentage points in low-readiness districts and by 21.7 percentage points in high-readiness districts (see points A‒C and D‒F in Fig 3-b, respectively) between 2001 and 2016. The DID model estimated that the gap between poor and nonpoor increased by 6.5 percentage points between low and high facility readiness areas during this period, which is the DIDID estimate (see the coefficient of ‘Nonpoor×High×2016’ under the column for facility delivery in Table 4). As these DID and DIDID estimates are statistically significant at p < 0.05, socioeconomic inequity increased in both low and high-readiness districts but faster in the high-readiness districts during the study period, resulting in a more inequitable distribution of facility delivery services. The DID and DIDID estimates were also not statistically significant for the treatment of complications, indicating a negligible differential change in equity by facility readiness in the treatment of complications.

### Differential changes in equity by access to private facilities in maternal health services use

**Fig 4:**
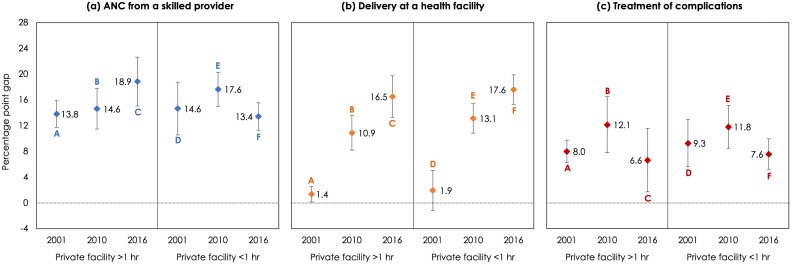
LPM coefficients showing changes in inequity in health services use by distance to private facilities^a,b^. ^**a**^ LPM coefficients from S4 Table 1 (in the supporting information) corresponding to the data points are as follows: A = Nonpoor; B = Nonpoor + Nonpoor×2010; C = Nonpoor + Nonpoor×2016; D = Nonpoor + Nonpoor×< 1hour; E = Nonpoor + Nonpoor×2010 + Nonpoor×<1 hour + Nonpoor×<1 hour×2010; and F = Nonpoor + Nonpoor×2016 + Nonpoor×< 1 hour + Nonpoor×< 1 hour×2016. ^b^ Error bars represent 95% confidence intervals.

From the respondent’s characteristics and the factors affecting maternal health services use, we observed that the availability of private health facilities had a statistically significant association with the use of maternal health services in 2016 (analysis not shown). For this reason, we examined how the poor–nonpoor gap in maternal health service use varied with the proximity of private health facilities, after controlling for all other relevant covariates, including facility readiness (see Fig 4). We found that for ANC, socioeconomic inequity increased by 5.1 percentage points in areas more than an hour away from private facilities (see points A‒C in Fig 4-a) but decreased by 1.2 percentage points in the areas closer to private facilities during the study period (see points D‒F in Fig 4-a). The DID model also estimated that socioeconomic inequity decreased by 6.3 percentage points between the areas with >1-hour and <1-hour distance from private facilities during this period, and this DIDID estimate was statistically significant at p < 0.05 (see the coefficient of ‘Nonpoor×<1 hour×2016’ under the column for ANC in S4 Table in the supporting information). We, therefore, conclude that socioeconomic inequity in ANC use decreased between 2001 and 2016 among women living <1 hour from a private facility.

For facility delivery, we can see from Fig 4-b that socioeconomic inequity increased similarly in both areas with >1-hour and <1-hour distance to private facilities (see Fig 4-b), and the estimated DIDID was not statistically significant (see the coefficient of ‘Nonpoor×<1 hour×2016’ under the column for facility delivery in S4 Table). For the treatment of delivery complications, socioeconomic inequity slightly decreased in both areas (see Fig 4-c), and the DID and DIDID estimates were also not statistically significant (see S4 Table).

We also examined how changes in socioeconomic equity differed by proximity to private health facilities and by facility readiness for maternal health service use, after controlling for relevant covariates. We found that the difference in changes in socioeconomic inequity between areas with >1-hour and <1-hour distance to private facilities, after accounting for facility readiness, remained not statistically significant for any maternal health service. See S5 Text in the supporting information for a detailed description of how the LPM coefficients were identified to measure equity, and S6 Table for the results of the regression models.

## Discussion

This study roughly covers the period 1998‒2016 to analyze how the use of key maternal health services has changed over time, focusing on equitable distribution in service use. During this period, the overall use of ANC, facility delivery, and treatment of delivery complications services increased steadily in Bangladesh. This finding is in line with the national health sector program evaluations and other studies [48–50]. Our analysis, however, indicated that the increase in uptake of key maternal health services, particularly for facility delivery, had not been uniform for all population groups. Other studies also found similar findings for the inequity in facility delivery for Bangladesh and other low- and middle-income countries in Asia and Africa [19,51–55]. Whether the specific pattern we observe (viz., supply-side readiness gains coinciding with widening socioeconomic inequity in facility delivery) is unique to Bangladesh or generalizable to other low- and middle-income countries remains an open question. The mechanisms we discuss below (low absolute facility readiness, persistent demand-side and affordability barriers, and rapid private-sector growth) have also been documented in other South Asian and sub-Saharan African settings, suggesting that the tension between supply-side expansion and equitable utilization may not be unique to Bangladesh.

A study on 74 low- and middle-income countries postulated that higher education and greater political commitment (measured as the government’s spending on health) were significantly associated with higher equity of reproductive and maternal health services use [51]. Most births in Bangladesh now take place among women in their 20s, where the largest improvement in years of schooling has taken place—between 2001 and 2017/18, the proportion of ever-married women of reproductive age having at least some secondary schooling more than doubled from 25% to 52%, and the proportion with no schooling declined from 47% to 17% [14,33]. This change has crucial implications for negotiating power within the family, for birth planning, for levels of awareness of maternal complications, for the potential to respond effectively to maternal complications, and for the ability to navigate the healthcare system [10]. With the initiation of the first sector-wide approach (SWAp) for the health sector in Bangladesh in 1998, there has been a considerable increase in the availability of, and access to, health facilities and expansion to maternal health care services through increasing health sector investment, augmenting human resources, and increasing availability of drugs and equipment to make the health facilities more functional [13]. The availability of private sector facilities also increased substantially, particularly after 2001—our analysis found that the availability of a private health facility within one hour of travel increased from 17% in 2001 to 68% in 2010 to 84% in 2016. Moreover, there has been steady macroeconomic growth and improvement in household economic conditions during the past two decades. The key question is why inequity in maternal health services use remained persistent (or, in one case, worsened) despite having all the elements needed to progress towards equitable service utilization in Bangladesh.

We believe several factors can explain the persistent inequity in maternal health services use in Bangladesh. First, our study indicated that high facility readiness was not associated with equitable use of maternal health services, contrary to existing research and recommendations from global health experts on strengthening health systems [56–60]. While estimating the district-level average of facility readiness for maternal health services in Bangladesh, we observed that the average facility readiness score was at a very low level in 2016 (0.322, meaning the health facilities had only 32% of their highest possible readiness level) and there has been a secular decline in average facility readiness index since 2001. Such a low level of readiness is perhaps the reason our analyses did not see a clear association between high facility readiness and equitable use of maternal health services. Our analyses indicated that socioeconomic inequity in all three maternal health services used continued to be significant in both low and high-facility-readiness districts and actually increased significantly faster in the districts with high readiness for facility delivery. We argue that the facility readiness of maternal health services in Bangladesh is too low to be associated with meaningful reductions in socioeconomic inequity. For example, normal delivery services are available in all district- and subdistrict-level public health facilities and private hospitals, more than half of the union-level facilities, and a third of the NGO clinics. The 2017 BHFS demonstrated that around 40% of facilities have a staff member trained in routine labor and delivery care or active management of the third stage of labor, 12% have guidelines related to basic or comprehensive emergency obstetric care, and there were acute shortages in essential life-saving drugs and commodities [36]. Overall, only 1% of facilities were found to have all of the 13 items (e.g., human resources, equipment, medicines) considered to be essential by the WHO to provide quality services [39], and only 11% of facilities had performed all seven basic signal functions for obstetric care in the last three months [36]. For ANC, only 4% of facilities are at the level of readiness necessary to provide quality ANC services, and there was no improvement in service readiness between 2014 and 2017, either for normal delivery or ANC at the facility level [36]. Universal access to and coverage of quality healthcare services is a critical element of the SDGs and a prerequisite for achieving equity in health [61–63]. With such low capacity and service readiness of the health system, in both public and private sectors, to provide critical maternal health services may partly explain the persistent inequity in service use in Bangladesh. A systematic review showed that the low readiness of health facilities contributed to inequities in skilled delivery care [64]. Even the effectiveness of health services innovations (viz., targeted service delivery, demand-side financing, and contracting out facilities) in improving equity in low and lower-middle-income countries was found to be contingent on the government’s capacity to enforce accountability mechanisms and provide adequate program inputs (financing and workforce) [65,66]. Demand-side financing schemes, in particular, were found to improve equitable skilled delivery care when facilities had higher service readiness [67,68].

Second, facility readiness alone cannot improve the equitable use of maternal health services unless the demand-side barriers (viz., personal, structural, and financial) to equitable health care are addressed. All the BMMS rounds explored why women did not seek medically-trained care during pregnancy and delivery and found that only 10% or less of the surveyed respondents reported low quality of care to be a reason for not delivering in a facility (for not seeking ANC or treatment for delivery complications, the rates are even lower) [31–33]. The major reason for not seeking care during pregnancy and delivery was the perceived absence of need (“not necessary”) along with social/cultural norms, followed by monetary constraints (e.g., cost of service was high or the household did not have the means to pay for service) and transport and access issues (e.g., facility too far or transport not available). As for-profit private hospitals tend to cluster in urban areas [69], the government has established (or upgraded) thousands of facilities (UHFWCs, USC/RDs, and CCs) at the community level, which was supposed to have a positive effect on maternal health services use. Our analysis, however, demonstrated that proximity to a public health facility had a statistically significant association with only facility delivery, and the association remained similar (i.e., change in regression coefficient was not significant) throughout the study period. One possible explanation for the public facilities not having the desired effect would be the lack of capacity to provide quality maternal health services—there were also no wide-scaled community mobilization and/or non-governmental facilitation in place to change the societal norms on maternal care-seeking, which were found to be effective in increase service use and improving equity in low- and middle-income countries in Asia and Africa [70–72].

Lastly, our analysis clearly demonstrated substantial growth of the private sector for maternal health services in Bangladesh, which is supported by the trend data from multiple rounds of Bangladesh Demographic and Health Surveys between 2000 and 2018 [14,73]. Currently, 32% of deliveries take place in a private facility, which is expected to rise to half of all deliveries by 2030 [74]. Our analysis showed that living closer (<1 hour of travel) to a private health facility was significantly associated with the use of maternal health services in 2016, and its association with facility delivery as well as treatment for delivery complications strengthened significantly between 2010 and 2016. Earlier studies indicated that low readiness and provision of services in the public sector could be the reason for such growth in the private sector, where wealthier and more educated women mostly rely on the private sector for reproductive and maternal health services [75–77]. Private hospitals also have performed markedly better than public facilities in terms of basic amenities (i.e., cleanliness, uninterrupted electricity, patient privacy, and toilet facilities for female patients) and basic laboratory diagnostic capacity (i.e., hemoglobin, urine protein, and urine glucose) [35,36], which would be another reason to attract clients who were willing to pay more for delivery services. The average delivery cost in a health facility increased from US $87 in 2001 to $204 in 2010 and stayed at the same level until 2016 in Bangladesh [31–33]. During this period, the cost of delivery in the private sector rose much faster than in the public sector—in 2001, the mean cost of delivery in a private facility was 1.2 times higher than the mean expenditures associated with deliveries at public facilities, which became 2.1 times higher in 2010 and 2.5 times higher in 2016. Private hospitals, however, had similar (or lower) maternal health service readiness to comparable public facilities (viz., DHs, UHCs, MCWCs) and performed considerably less than the comparable public facilities in terms of availability of trained providers and carrying out the routine quality control/quality assurance activities [36]. Both the substantial growth of the private sector and their high associated with low-quality care are major impediments to achieving equity in maternal health services. This could be a reason why we saw a significant reduction in inequity for a relatively low-cost service like ANC in the areas near the private sector, but not for facility delivery or treatment of complications.

Based on the findings of this study and review of the experiences of low- and middle-income countries that are implementing universal health coverage to improve equity in maternal health services utilization, this paper outlines the following policy recommendations to be pursued in the short- and medium-term:

1. *Test and expand effective community mobilization and social protection schemes for health:* The equiplots in our analysis indicated only a modest reduction in the gap between the poorest and the richest quintiles for ANC and treatment of delivery complications, but a notable increase in the gap between the poorest and the richest wealth quintiles for facility delivery. The government has various social protection schemes targeted at specific vulnerable population groups, which need to be linked with maternal health services to provide financial protection for the poor against catastrophic health expenditures and improve equity. The government and civil society should also explore community mobilization, and behavior change communication activities to effectively address the social and cultural barriers to maternal health service use, particularly targeting the vulnerable population groups that are the least likely to use maternal health services.
2. *Ensure service readiness at all levels of health facilities:* In order to observe the role of service readiness on equity in maternal health services use, the service readiness level has to be improved considerably from the current level. The Government of Bangladesh needs to focus on key reproductive health services with evidence to have a high impact in improving maternal health (e.g., full-component ANC for pregnant women at all facilities and delivery care with the management of delivery complications at strategically located facilities to minimize access barriers) [78]. To increase the service capacity and readiness at the facilities, the government needs to ensure an adequate supply of drugs and medical commodities (guidelines, equipment) along with building, allocating, and retaining the capacity of skilled providers in public health facilities of all levels [74]. In order to ensure that all health sector stakeholders, including the private sector, adhere to policies, procedures, and quality standards of health services delivery, the government needs to take a stronger governance and stewardship role.
3. *Increase the share of the public sector for delivery services:* Evidence from South and Southeast Asian countries indicated that increasing the public healthcare provisions can accelerate progress towards equitable coverage of health services [79]. Major financial and institutional reforms will need to be implemented to improve service readiness and quality at the primary level, as it is more efficient to increase utilization of inpatient and outpatient health services at lower levels of the service delivery system [80]. In addition, more rational planning in the proliferation of health facilities is needed—we recommend that the government should start with expanding the provision of deliveries in public sector health facilities by operationalizing 4,546 Union Health Centers across the country, each serving on average 25,000 population, for conducting normal deliveries and referral of complicated pregnancies to higher facilities.

### Study limitations

This study’s limitations are largely related to the nature of the data used. First, all analyses relied on women’s recall of details about the ANC and delivery care that they received for a live birth up to three years preceding the survey. Women’s responses were classified into pre-specified survey response options, and there is a possibility of recall bias in their responses despite rigorous training for the field interviewers and several layers of data quality checks in place. Reporting of delivery complications were ‘perceived complications,’ and no medical diagnosis was carried out to ascertain the validity of the responses. Also, data were not collected about the care received by women who experienced non-live birth outcomes such as miscarriages, stillbirths, and induced abortions. Second, linking structural/ programmatic variables (e.g., the readiness of health facilities) to surveyed households at the sampled cluster level was not possible since the BHFS/SPA and BMMS surveys were sampled independently. Instead, we linked facilities and households at the lowest possible geographic unit, which is at the district level. This level of linking provides information on the ‘existing service environment’ for the BMMS respondents rather than the readiness of facilities where delivery care was actually sought (linking women to individual facilities would be problematic conceptually anyway because a lot of women don’t use facilities for delivery care). Third, the data availability of potential structural/ programmatic covariates is not uniform across the BHFS/SPA survey rounds. For example, the 1999‒2000 Bangladesh SPA did not collect information from secondary-level public health facilities such as government district hospitals, for which the bed occupancy rate of district hospitals served as a proxy for service efficiency/readiness and imputed readiness scores for 1999‒2000 based on regression outputs for 2011 and 2017. Fourth, because facility readiness was measured contemporaneously rather than as a discretely timed intervention, the readiness–equity association may be confounded by concurrent secular changes during 2001–2016, including successive national health-sector programs, the rapid expansion of the private sector, and broad improvements in women’s education and household economic conditions. These could independently affect both facility readiness and equitable utilization, and our design cannot fully disentangle these time-varying influences. Fifth, because the analysis pools three independent repeated cross-sections, the composition of respondents (e.g., age, education, parity, and urban–rural residence) shifted across survey rounds and may have shifted differentially across readiness groups. Although these characteristics are adjusted for in the regression models, residual compositional differences could still influence the observed trends, and we did not estimate transition-based models. Sixth, because facility readiness is measured contemporaneously at the district-year level rather than as a single intervention with a common adoption date, and because districts can move between readiness categories across rounds, conventional difference-in-differences diagnostics, such as pre-treatment parallel-trends tests, event-study plots, anticipation or placebo tests, and staggered-adoption estimators, are not directly applicable. Accordingly, we present our findings as differential associations in trends rather than causal effects, and we did not undertake additional sensitivity analyses using alternative readiness thresholds, control groups, or stratified subsamples (e.g., by urban–rural residence, administrative division, baseline poverty, or public/private facility mix), which remain valuable directions for future research. Finally, our readiness measure captures structural capacity only; we were unable to supplement it with process-quality or patient-experience indicators, which were not consistently available across the linked survey rounds, so we cannot empirically establish that structural readiness translated into the quality of care actually experienced by women.

The data sources used for this study did not collect information on ideation and norms in maternal health services use, which remains a gap in this analysis. Further research is needed to understand beliefs and social/cultural norms around pregnancy, delivery, and postpartum, as well as their relation to service utilization and quality of care in health facilities. Despite an ongoing global movement for measuring and improving respectful care, only a few studies have looked into respectful maternity care in Bangladesh [81,82]. Further research is also needed to understand respectful maternity care as an important factor in influencing the use of services.

## Conclusions

Lessons from Bangladesh’s maternal and child health services delivery and service utilization during the last two decades have important implications for policymakers and public health researchers in low- and middle-income countries [83]. In this study, we attempted to provide a comprehensive analysis of socioeconomic inequities in the use of essential maternal health services in Bangladesh and examined how socioeconomic inequities varied with supply-side factors during 2001‒2016. This study found that significant progress has been made in Bangladesh in increasing the use of essential maternal health services. However, it also found persistent socioeconomic inequities in the use of all three key maternal health services, and that the inequity in facility delivery worsened over time. This finding is pertinent to understanding the stagnation of maternal mortality decline in recent years in Bangladesh, as improving equity in maternal health service use is critical to reducing maternal mortality [12,84–87]. To our knowledge, this is the first examination of the role of supply-side factors on the use of maternal health services and a systematic investigation of the role of facility readiness on maternal health equity in Bangladesh using large-scale, comprehensive, nationally representative data. The empirical evidence and policy measures presented here provide a way forward for policymakers to provide equitable care for safe delivery services in Bangladesh.

## Supporting information

S1 Table Definitions for obstetric and newborn care readiness indicators, Bangladesh 1999–2017. (PDF)

S2 Table District averages of facility readiness score, Bangladesh 2000, 2011, and 2017. (PDF)

S3 Table Differential changes in socioeconomic equity by access to private facilities, Bangladesh 2001–2016. (PDF)

S4 Table LPM estimates of differential changes in socioeconomic equity by facility readiness and distance to private facility, Bangladesh 2001–2016. (PDF)

S1 Text Derivation of the LPM coefficients used to measure equity. (PDF)

S2 Text Derivation of the combined facility-readiness and private-facility model. (PDF)
