# Supplementary material for: Socioeconomic equity in maternal health services use in Bangladesh: The role of service readiness in health facilities during the period 2001–2016
Source: PLoS One. 2026 Jul 30;21(7):e0354897. doi: 10.1371/journal.pone.0354897 (PMC13422858; doi:10.1371/journal.pone.0354897)
Supplement: S1 Table — (PDF) [file pone.0354897.s001.pdf]

S1 Table. Definitions for obstetric care readiness indicators, Bangladesh 1999–2017

Domain/Indicator Name	Definition	Data availability		
		2000	2011	2017
**Domain A: Comprehensive emergency obstetric care signal functions**				
Parenteral administration of antibiotics	Facility performed this signal function for emergency obstetric care at least once during the three months before the assessment	Y[1]	Y[2]	Y
Parenteral administration of uterotonic drugs/ oxytocin	Facility performed this signal function for emergency obstetric care at least once during the three months before the assessment	Y	Y[3]	Y
Parenteral administration of anticonvulsants for hypertensive disorders of pregnancy	Facility performed this signal function for emergency obstetric care at least once during the three months before the assessment	Y	Y[4]	Y
Assisted vaginal delivery	Facility performed this signal function for emergency obstetric care at least once during the three months before the assessment	Y	Y[5]	Y
Removal of retained products	Facility performed this signal function for emergency obstetric care at least once during the three months before the assessment	Y	Y[6]	Y
Cesarean section	Facility performed this signal function for emergency obstetric care at least once during the three months before the assessment	Y	Y	Y
Blood transfusion	Facility performed this signal function for emergency obstetric care at least once during the three months before the assessment	Y	Y	Y
**Domain B: Newborn signal functions and immediate care**				
Neonatal resuscitation	Facility performed neonatal resuscitation at least once during the three months before the assessment	Y	Y[7]	Y
**Domain C: General requirements**				
Electricity	Facility is connected to a central power grid, or the facility had a functioning generator.	Y	Y	Y
Improved water source	Facility has an improved water source available. For most countries, this means that water is piped into the facility or onto facility grounds, or else water comes from a public tap or standpipe, a tube well or borehole, a protected dug well, protected spring, rainwater, or bottled water, and the outlet from this source is within 500 meters of the facility.	Y	Y	Y
Improved sanitation	Facility has a functioning flush or pour-flush toilet, a ventilated improved pit latrine, or composting toilet.	Y	Y	Y

1 For the 9 signal functions below, timeframe (last 3 months) not considered as data not available
2 Gentamycin is available as injections only—the rest are for both parenteral/oral administration
3 Continuously available in the past 30 days
4 Continuously available in the past 30 days; diazepam/antihypertensive medications available are usually orally administered
5 Regularly used vacuum extractor/forceps
6 Regularly used manual vacuum aspirator/suction bulb
7 Regularly used Resuscitation bag for newborn

Capacity for laboratory tests	Facility has a functioning laboratory to conduct basic diagnostic tests.	Y[8]	Y[9]	Y[10]
24/7 Skilled birth attendance	Provider of delivery care available on-site or on-call 24 hours/day, with observed duty schedule.	Y	Y	Y
Emergency transport	The facility had a functioning ambulance or other vehicle for emergency transport.	Y	Y	Y[11]
Delivery bed	At least one bed available and observed in delivery area.	Y[12]	Y[13]	Y
**Domain D: Equipment**				
Sterilization equipment	Facility reports that some instruments are processed in the facility and the facility has a functioning electric dry heat sterilizer, a functioning electric autoclave.	Y	Y	Y
Delivery pack	Delivery pack OR cord clamp, episiotomy scissors, scissors/lade to cut cord, suture material with need, and needle holder all available in delivery area.	Y	Y[14]	Y
Infant scale	Infant scale observed and functioning in delivery area.	Y	Y	Y
Adult scale	Weighing scale observed and functioning in delivery area.	Y	Y	Y
Blood pressure apparatus	Manual or digital blood pressure apparatus observed and functioning in delivery area.	Y	Y	Y
Stethoscope	Stethoscope observed and functioning in delivery area.	Y	Y	Y
**Domain E: Medicines and commodities**				
Injectable antibiotic	Injectable antibiotics observed in delivery area (i.e., at “service site”) and at least one dose valid.	Y[15]	Y[16]	Y
Injectable uterotonic	Oxytocin observed in delivery area with at least one dose valid.	Y[17]	Y	Y
Magnesium sulfate	Magnesium sulfate available in delivery area with at least one dose valid.	Y	Y	Y
IV solution with infusion set	IV solution with infusion set available in delivery area with at least one set valid.	Y[18]	Y	Y
Chlorhexidine for cord cleaning	Chlorhexidine solution (4%) for umbilical cord cleaning available in delivery area, with at least one dose valid.	Y[19]	Y[20]	Y
Antibiotic eye ointment for newborn	Tetracycline eye ointment for newborns available in delivery area and at least one dose valid.	Y[21]	Y[22]	Y

[8] Routine blood and urine tests
[9] CBC, anemia, urine for R/M/E, blood glucose, pregnancy tests
[10] Any hemoglobin, any blood glucose, any urine chemistry testing
[11] Either the facility has onsite or has access to
[12] Availability of beds in facilities providing maternal services considered
[13] Availability of beds in facilities providing maternal services considered
[14] Delivery kit (instruments, supplies) available, complete or incomplete
[15] Cloxacillin, Ampicillin, Gentamycin and Metronidazole considered (not Amoxycillin)
[16] Except Gentamycin, others are available both in injectable and tablet/capsule forms
[17] Syntocenon, Ergometrin considered (Misoprostol data not collected)
[18] IV fluid only (infusion set data not collected)
[19] Gentian violet considered (Chlorhexidine data not collected)
[20] Clorohexidine or cetrimide solution 1 litre
[21] Chloramphenicol considered (Tetracycline data not collected)
[22] Eye drops or ointment for newborn
