# Supplementary material for: Socioeconomic equity in maternal health services use in Bangladesh: The role of service readiness in health facilities during the period 2001–2016
Source: PLoS One. 2026 Jul 30;21(7):e0354897. doi: 10.1371/journal.pone.0354897 (PMC13422858; doi:10.1371/journal.pone.0354897)
Supplement: S2 Table — (PDF) [file pone.0354897.s002.pdf]

S2 Table. District averages of facility readiness score, Bangladesh 1999–2000, 2011, and 2017

District Name	BHFS/SPA Survey rounds		
	2000	*2011*	*2017*
Bagerhat	0.543	0.271	0.314
Bandarban	0.562	0.741	0.31
Barguna	0.633	0.195	0.294
Barisal	0.594	0.181	0.308
Bhola	0.571	0.844	0.289
Bogra	0.675	0.219	0.327
Brahmanbaria	0.462	0.261	0.289
Chanpur	0.607	0.297	0.356
Chittagong	0.478	0.201	0.318
Chuadanga	0.549	0.741	0.389
Comilla	0.515	0.225	0.329
Cox's Bazar	0.476	0.313	0.461
Dhaka	0.464	0.338	0.401
Dinajpur	0.532	0.268	0.372
Faridpur	0.565	0.194	0.304
Feni	0.57	0.682	0.372
Gaibandha	0.421	0.184	0.231
Gazirpur	0.525	0.178	0.362
Gopalganj	0.481	0.657	0.231
Habiganj	0.524	0.296	0.344
Joypurhat	0.697	0.252	0.353
Jamalpur	0.55	0.18	0.341
Jessore	0.624	0.184	0.311
Jhalokati	0.581	0.282	0.279
Jhenaidaha	0.615	0.178	0.308
Khagrachhari	0.654	0.436	0.498
Khulna	0.652	0.162	0.311
Kishoreganj	0.428	0.296	0.325
Kurigram	0.578	0.206	0.35
Kushtia	0.488	0.162	0.318
Lakshmipur	0.503	0.238	0.308
Lalmonirhat	0.503	0.252	0.298
Madaripur	0.481	0.69	0.27
Magura	0.639	0.422	0.298
Manikganj	0.535	0.487	0.327
Meherpur	0.521	0.556	0.308
Maulvi Bazar	0.506	0.251	0.304
Munshiganj	0.568	0.214	0.359
Mymensingh	0.556	0.211	0.258
Naogaon	0.668	0.287	0.299
Narail	0.564	0.889	0.276
Naratanganj	0.576	0.647	0.322
Narsingdi	0.516	0.321	0.228
Natore	0.371	0.198	0.314
Nawabganj	0.488	0.783	0.29
Netrokona	0.448	0.348	0.27
Nilphamari	0.58	0.778	0.284
Noakhali	0.514	0.905	0.328
Pabna	0.588	0.248	0.314

Panchagarh	0.573	0.171	0.288
Patuakhali	0.676	0.782	0.382
Pirojpur	0.566	0.606	0.316
Rajshahi	0.545	0.815	0.326
Rajbari	0.511	0.667	0.299
Rangamati	0.664	0.233	0.314
Rangpur	0.579	0.218	0.406
Shariatpur	0.628	0.203	0.402
Satkhira	0.584	0.251	0.297
Siraganj	0.675	0.116	0.345
Sherpur	0.495	0.571	0.311
Sunamganj	0.559	0.172	0.3
Sylhet	0.548	0.36	0.305
Tangail	0.507	0.225	0.282
Thakurgaon	0.618	0.173	0.279
**Total**	**0.544**	**0.341**	**0.322**

Note: Columns show the BHFS/SPA facility-survey rounds (1999-2000, 2011, and 2017), linked to the BMMS 2001, 2010, and 2016 household data, respectively.
