# Supplementary material for: Socioeconomic equity in maternal health services use in Bangladesh: The role of service readiness in health facilities during the period 2001–2016
Source: PLoS One. 2026 Jul 30;21(7):e0354897. doi: 10.1371/journal.pone.0354897 (PMC13422858; doi:10.1371/journal.pone.0354897)
Supplement: S3 Table — (PDF) [file pone.0354897.s003.pdf]

S4 Table. Differential changes in socioeconomic equity by access to private facilities, Bangladesh 2001–2016

Background Characteristics	Skilled ANC	Facility delivery	Complication treatment
Woman's age at birth (reference category: <18 years)			
18-24	0.039**	0.035**	0.054**
25-29	0.085**	0.091**	0.108**
30-34	0.113**	0.127**	0.141**
35-39	0.088**	0.134**	0.159**
40-49	0.079**	0.135**	0.165**
Parity (reference category: 1)			
2-3	-0.073**	-0.098**	-0.063**
4 or more	-0.161**	-0.169**	-0.100**
Place of residence (reference category: urban)			
Rural	-0.079**	-0.083**	-0.059**
Women's education (reference category: no schooling)			
Any primary	0.096**	0.016**	0.060**
Secondary incomplete	0.229**	0.114**	0.164**
Secondary complete or higher	0.346**	0.317**	0.280**
Time to reach the nearest public health facility (reference category: >1 hour)			
<1 hour	0.058**	0.023**	0.038**
Facility readiness (reference category: low readiness)			
High readiness	-0.021*	-0.005	-0.021*
Socioeconomic status (reference category: poor)			
Nonpoor	0.138**	0.014*	0.080**
Survey round (reference category: 2001)			
2010	0.013	0.017*	0.043*
2016	0.142**	0.113**	0.142**
Interaction between socioeconomic status and survey round			
Nonpoor×2010	0.008	0.095**	0.041†
Nonpoor×2016	0.051*	0.152**	-0.014
Time to reach the nearest private health facility (reference category: >1 hour)			
<1 hour	0.008	-0.009	0.012
Interaction between time to reach the nearest private health facility and survey round			
<1 hour×2010	0.008	0.030*	-0.008
<1 hour×2016	0.055*	0.109**	0.032
Interaction between socioeconomic status and time to reach the nearest private health facility			
Nonpoor×<1 hour	0.008	0.006	0.013
Interaction between socioeconomic status, time to reach private facility, and survey round			
Nonpoor×<1 hour×2010	0.022	0.016	-0.016
Nonpoor×<1 hour×2016	-0.063*	0.005	-0.003
Constant	0.287**	0.114**	0.123**
Nonpoor×2010 + Nonpoor×<1 hour×2010	0.030	0.112**	0.025
Nonpoor×2016 + Nonpoor×<1 hour×2016	-0.012	0.156**	-0.017
Nonpoor + Nonpoor×2010	0.146**	0.109**	0.121**
Nonpoor + Nonpoor×2016	0.189**	0.165**	0.066*
Nonpoor + Nonpoor×<1 hour	0.146**	0.019	0.093**
Nonpoor + Nonpoor×2010 + Nonpoor×<1hr + Nonpoor×<1hr×2010	0.176**	0.131**	0.118**
Nonpoor + Nonpoor×2016 + Nonpoor×<1hr + Nonpoor×<1hr×2016	0.134**	0.176**	0.076**

Note: ** $p<0.001$; * $p<0.05$; † $p<0.10$
