# Supplementary material for: Socioeconomic equity in maternal health services use in Bangladesh: The role of service readiness in health facilities during the period 2001–2016
Source: PLoS One. 2026 Jul 30;21(7):e0354897. doi: 10.1371/journal.pone.0354897 (PMC13422858; doi:10.1371/journal.pone.0354897)
Supplement: S4 Table — (PDF) [file pone.0354897.s004.pdf]

S6 Table. LPM estimates of differential changes in socioeconomic equity by facility readiness and distance to private facility in maternal health service use, Bangladesh 2001–2016

Background Characteristics	Skilled ANC	Facility delivery	Complication treatment
Woman's age at birth (reference category: <18 years)			
18-24	0.039**	0.035**	0.054**
25-29	0.085**	0.091**	0.108**
30-34	0.113**	0.126**	0.141**
35-39	0.088**	0.134**	0.159**
40-49	0.079**	0.135**	0.165**
Parity (reference category: 1)			
2-3	-0.073**	-0.098**	-0.063**
4 or more	-0.161**	-0.169**	-0.100**
Place of residence (reference category: urban)			
Rural	-0.077**	-0.082**	-0.059**
Women's education (reference category: no schooling)			
Any primary	0.096**	0.016**	0.059**
Secondary incomplete	0.229**	0.114**	0.163**
Secondary complete or higher	0.346**	0.318**	0.280**
Time to reach the nearest public health facility (reference category: >1 hour)			
<1 hour	0.059**	0.024**	0.037**
Facility readiness (reference category: low readiness)			
High readiness	-0.014	-0.007	0.002
Survey round (reference category: 2001)			
2010	0.012	0.010	0.045*
2016	0.140**	0.122**	0.169**
Interaction between facility readiness and survey round			
High×2010	0.005	0.020	-0.005
High×2016	0.006	-0.031	-0.092†
Socioeconomic status (reference category: poor)			
Nonpoor	0.146**	0.015†	0.086**
Interaction between socioeconomic status and survey round			
Nonpoor×2010	0.000	0.099**	0.039
Nonpoor×2016	0.054*	0.143**	-0.029
Time to reach the nearest private health facility (reference category: >1 hour)			
<1 hour	0.013	-0.018*	0.022
Interaction between time to reach the nearest private health facility and survey round			
<1 hour×2010	0.008	0.038*	-0.017
<1 hour×2016	0.051	0.129**	0.007
Interaction between socioeconomic status and time to reach the nearest private health facility			
Nonpoor×<1 hour	0.006	0.010	0.012
Interaction among socioeconomic status, time to reach the nearest private facility, and survey round			
Nonpoor×<1 hour×2010	0.016	-0.004	-0.020
Nonpoor×<1 hour×2016	-0.072*	-0.013	0.004
Interaction between socioeconomic status and facility readiness			
Nonpoor×High	-0.025	-0.005	-0.020
Interaction between facility readiness and time to reach the nearest private facility			
High×<1 hour	-0.015	0.026*	-0.035
Interaction among socioeconomic status, facility readiness, and time to reach the nearest private facility			
Nonpoor×High×<1 hour	0.006	-0.008	0.000
Interaction among socioeconomic status, facility readiness, and survey round			
Nonpoor×High×2010	0.025	-0.012	0.007

Nonpoor×High×2016	-0.013	0.030	0.054
Interaction among facility readiness, time to reach the nearest private facility, and survey round			
High×<1 hour×2010	-0.006	-0.021	0.031
High×<1 hour×2016	0.008	-0.057	0.085
Interaction among socioeconomic status, facility readiness, time to reach the nearest private facility, and round			
Nonpoor×High×<1 hour×2010	0.021	0.051	0.012
Nonpoor×High×<1 hour×2016	0.032	0.051	-0.022
Constant	0.284**	0.113**	0.117**

Note: ** $p<0.001$; * $p<0.05$; † $p<0.10$
