# Supplementary material for: Socioeconomic equity in maternal health services use in Bangladesh: The role of service readiness in health facilities during the period 2001–2016
Source: PLoS One. 2026 Jul 30;21(7):e0354897. doi: 10.1371/journal.pone.0354897 (PMC13422858; doi:10.1371/journal.pone.0354897)
Supplement: S1 Text — (PDF) [file pone.0354897.s005.pdf]

S3 Text: Derivation of the LPM coefficients to measure equity

Equation (2) presented in the Methods section has the following specification:

$$Y_{ijt} = \beta_0 + \beta_1 S_{it} + \beta_2 T_t + \beta_3 S_{it} \cdot T_t + \beta_4 Z_{jt} + \beta_5 Z_{jt} \cdot S_{it} + \beta_6 Z_{jt} \cdot T_t + \beta_7 Z_{jt} \cdot S_{it} \cdot T_t + \beta_8 X_{ijt} + \varepsilon_{ijt} \quad \ldots\ldots (2)$$

where, Y_{ijt} is the outcome of interest for individual *i* who lives in district j at time *t*. S takes the value of 1 if the woman's socioeconomic status is nonpoor and 0 if poor. T is a vector of time dummies to represent the BMMS survey rounds. For the illustration of how to derive LPM coefficients in this appendix, the time dummy takes the value of 1 if the BMMS survey round is 2016 and 0 if 2001. A similar derivation process is used to derive the LPM coefficients for 2001 and 2010. Z is an indicator variable that takes the value of 1 if the woman lives in a district with high facility readiness and 0 otherwise. X are the control variables.

For nonpoor (i.e., S_{it}= 1), equation (2) becomes

$$Y_{ijt} = \beta_0 + \beta_1 + (\beta_2 + \beta_3) T_t + (\beta_4 + \beta_5) Z_{jt} + \beta_6 Z_{jt} \cdot T_t + \beta_7 Z_{jt} \cdot T_t \quad \ldots\ldots (3)$$

For poor (i.e., S_{it}=0), equation (2) becomes

$$Y_{ijt} = \beta_0 + \beta_2 T_t + \beta_4 Z_{jt} + \beta_6 Z_{jt} \cdot T_t \quad \ldots\ldots (4)$$

Therefore, socioeconomic inequity can be measured by subtracting (4) from (3),

$$Inequity = \beta_1 + \beta_3 T_t + \beta_5 Z_{jt} + \beta_7 Z_{jt} \cdot T_t \quad \ldots\ldots (5)$$

For districts with low facility readiness (i.e., Z_{jt}= 0), equation (5) at baseline (i.e., T_t= 0), becomes

$$Inequity = \beta_1 \quad \ldots\ldots (6)$$

For districts with low facility readiness (i.e., Z_{jt}= 0), equation (5) at endline (i.e., T_t= 1), becomes

$$Inequity = \beta_1 + \beta_3 \quad \ldots\ldots (7)$$

For districts with high facility readiness (i.e., Z_{jt}= 1), equation (5) at baseline (i.e., T_t= 0), becomes

$$Inequity = \beta_1 + \beta_5 \quad \ldots\ldots (8)$$

For districts with high facility readiness (i.e., Z_{jt}= 1), equation (5) at endline (i.e., T_t= 1), becomes

$$Inequity = \beta_1 + \beta_3 + \beta_5 + \beta_7 \quad \ldots\ldots (9)$$

The change in socioeconomic inequity in low facility readiness districts during the study period can be measured as: (7) – (6) = $[\beta_1 + \beta_3] - \beta_1 = \beta_3$ ……. (10)

The change in socioeconomic inequity in high facility readiness districts during the study period can be measured as: (9) – (8) = $[\beta_1 + \beta_3 + \beta_5 + \beta_7] - [\beta_1 + \beta_5] = \beta_3 + \beta_7$ ……. (11)

Therefore, the difference in the changes in socioeconomic inequity between high and low facility readiness districts during the study period can be measured as follows: (11) – (10) = $[\beta_3 + \beta_7] - \beta_3 = \beta_7$.
