# Supplementary material for: Socioeconomic equity in maternal health services use in Bangladesh: The role of service readiness in health facilities during the period 2001–2016
Source: PLoS One. 2026 Jul 30;21(7):e0354897. doi: 10.1371/journal.pone.0354897 (PMC13422858; doi:10.1371/journal.pone.0354897)
Supplement: S2 Text — (PDF) [file pone.0354897.s006.pdf]

S5 Text. Derivation of the combined facility readiness and access to private health facility model

To examine how socioeconomic equity changed by facility readiness and distance to a private facility, equation (2) presented in the Methods section was extended as follows:

$$Y_{ijt} = \beta_0 + \beta_1 S_{it} + \beta_2 T_t + \beta_3 S_{it} \cdot T_t$$
$$+\beta_4 Z_{jt} + \beta_5 Z_{jt} \cdot S_{it} + \beta_6 Z_{jt} \cdot T_t + \beta_7 Z_{jt} \cdot S_{it} \cdot T_t$$
$$+\beta_8 P_{jt} + \beta_9 P_{jt} \cdot S_{it} + \beta_{10} P_{jt} \cdot T_t + \beta_{11} P_{jt} \cdot S_{it} \cdot T_t + \beta_{12} Z_{jt} \cdot P_{jt} \cdot S_{it} \cdot T_t + \beta_{13} X_{ijt} + \varepsilon_{ijt} \quad (12)$$

where, Y_{ijt} is the outcome of interest for individual *i* who lives in district *j* at time *t*. S takes the value of 1 if the woman's socioeconomic status is nonpoor and 0 if poor. T is the time dummy that takes the value of 1 if the BMMS survey round is 2010 (or 2016) and 0 if 2001. Z is an indicator variable that takes the value of 1 if the woman lives in a district with high facility readiness and 0 otherwise. P is also an indicator variable that takes the value of 1 if the woman lives in a cluster with <1 hour distance from the nearest private facility and 0 otherwise. X are the control variables.

For nonpoor (i.e., S=1), equation (12) becomes

$$Y_{ijt} = \beta_0 + \beta_1 + \beta_2 T_t + \beta_3 T_t + \beta_4 Z_{jt} + \beta_5 Z_{jt} + \beta_6 Z_{jt} \cdot T_t + \beta_7 Z_{jt} \cdot T_t$$
$$+\beta_8 P_{jt} + \beta_9 P_{jt} + \beta_{10} P_{jt} \cdot T_t + \beta_{11} P_{jt} \cdot T_t + \beta_{12} Z_{jt} \cdot P_{jt} \cdot T_t + \varepsilon_{ijt} \quad \ldots. (13)$$

For poor (S=0), equation (12) becomes

$$Y_{ijt} = \beta_0 + \beta_2 T_t + \beta_4 Z_{jt} + \beta_6 Z_{jt} \cdot T_t + \beta_8 P_{jt} + \beta_{10} P_{jt} \cdot T_t \quad \ldots. (14)$$

Therefore, socioeconomic inequity can be measured by subtracting (14) from (13),

$$\text{Inequity} = \beta_1 + \beta_3 T_t + \beta_5 Z_{jt} + \beta_7 Z_{jt} \cdot T_t + \beta_9 P_{jt} + \beta_{11} P_{jt} \cdot T_t + \beta_{12} Z_{jt} \cdot P_{jt} \cdot T_t \quad \ldots. (15)$$

For low readiness (Z=0), far private facilities (P=0) at baseline (T=0):

Inequity = β_1

For low readiness (Z=0), far private facilities (P=0) at endline (T=1):

Inequity = $\beta_1 + \beta_3$

For high readiness (Z=1), far private facilities (P=0) at baseline (T=0):

Inequity = $\beta_1 + \beta_5$

For high readiness (Z=1), far private facilities (P=0) at endline (T=1):

Inequity = $\beta_1 + \beta_3 + \beta_5 + \beta_7$

For low readiness (Z=0), close private facilities (P=1) at baseline (T=0):

Inequity = $\beta_1 + \beta_9$

For low readiness (Z=0), close private facilities (P=1) at endline (T=1):

Inequity = $\beta_1 + \beta_3 + \beta_9 + \beta_{11}$

For high readiness (Z=1), close private facilities (P=1) at baseline (T=0):

Inequity = $\beta_1 + \beta_5 + \beta_9$

For high readiness (Z=1), close private facilities (P=1) at endline (T=1):

Inequity = $\beta_1 + \beta_3 + \beta_5 + \beta_7 + \beta_9 + \beta_{11} + \beta_{12}$

Change in inequity over time for high readiness (Z=1), close private facilities (P=1) = $\beta_3 + \beta_7 + \beta_{11} + \beta_{12}$

Change in inequity over time for low readiness (Z=0), close private facilities (P=1) = $\beta_3 + \beta_{11}$

Change in inequity over time for high readiness (Z=1), far private facilities (P=0) = $\beta_3 + \beta_7$

Change in inequity over time for low readiness (Z=0), far private facilities (P=0) = β_3

Therefore, the difference in the changes in socioeconomic inequity between the areas with >1 hour and <1 hour distance to private facilities as well as low and high facility readiness districts during the study period can be measured as follows:

$$[(\beta_3 + \beta_7 + \beta_{11} + \beta_{12}) - (\beta_3 + \beta_{11})] - [(\beta_3 + \beta_7) - \beta_3] = \beta_{12}.$$
