# Supplementary material for: Socioeconomic equity in maternal health services use in Bangladesh: The role of service readiness in health facilities during the period 2001–2016
Source: PLoS One. 2026 Jul 30;21(7):e0354897. doi: 10.1371/journal.pone.0354897 (PMC13422858; doi:10.1371/journal.pone.0354897)
Supplement: Attachment — Submitted filename: Response_to_Reviewers_R1.docx [file pone.0354897.s008.docx]

**Authors’’ response to Editorial and Reviewers’ comments**

**Manuscript ID:** PONE-D-25-54631

**Editorial Office**

1. Please ensure that your manuscript meets PLOS ONE's style requirements, including those for file naming. The PLOS ONE style templates can be found at
   <https://journals.plos.org/plosone/s/file?id=wjVg/PLOSOne_formatting_sample_main_body.pdf> and <https://journals.plos.org/plosone/s/file?id=ba62/PLOSOne_formatting_sample_title_authors_affiliations.pdf>.

**Authors’ response:** We have formatted the manuscript, tables, figures, and file names in line with the PLOS ONE style templates for the main body and for the title/authors/affiliations, and have ensured the submission meets PLOS ONE's style and file-naming requirements.

2. Thank you for stating the following financial disclosure: [This publication was produced with the support of the United States Agency for International Development (USAID) under the terms of USAID’s Data for Impact (D4I) associate award no. 7200AA18LA00008. We are grateful to the Carolina Population Center and MCH Department of the Gillings School of Global Public Health, whose funding and financial support have made this possible. Views expressed herein do not necessarily reflect the views of the U.S. Government or USAID.]. Please state what role the funders took in the study. If the funders had no role, please state: ""The funders had no role in study design, data collection and analysis, decision to publish, or preparation of the manuscript."" If this statement is not correct you must amend it as needed. Please include this amended Role of Funder statement in your cover letter; we will change the online submission form on your behalf.

**Authors’ response:** We added “The funders had no role in study design, data collection and analysis, decision to publish, or preparation of the manuscript.” in the financial disclosure.

3. Please note that your Data Availability Statement is currently missing the DOI/accession number of each dataset OR a direct link to access each database. If your manuscript is accepted for publication, you will be asked to provide these details on a very short timeline. We therefore suggest that you provide this information now, though we will not hold up the peer review process if you are unable.

**Authors’ response:** Data Availability Statement updated by providing the required information.

4. Please include your full ethics statement in the ‘Methods’ section of your manuscript file. In your statement, please include the full name of the IRB or ethics committee who approved or waived your study, as well as whether or not you obtained informed written or verbal consent. If consent was waived for your study, please include this information in your statement as well.

**Authors’ response:** Included on p.7, lines 144–151.

5. Please include captions for your Supporting Information files at the end of your manuscript, and update any in-text citations to match accordingly. Please see our Supporting Information guidelines for more information: <http://journals.plos.org/plosone/s/supporting-information>.

**Authors’ response:** We have added a "Supporting Information" section at the end of the manuscript (pp.34–35, lines 832–840).

6. If the reviewer comments include a recommendation to cite specific previously published works, please review and evaluate these publications to determine whether they are relevant and should be cited. There is no requirement to cite these works unless the editor has indicated otherwise.

**Authors’ response:** Not applicable.

**Reviewer 1**

1. Figure 1 presents overall national trends in maternal health service utilisation across survey years but does not display wealth-stratified estimates. While wealth-quintile–specific patterns are presented elsewhere (e.g., Fig 2 and the Supplementary Information), statements referring to widening or narrowing gaps between the poorest and richest quintiles should not be attributed to Fig 1. The text would benefit from explicitly referencing the appropriate wealth-disaggregated figure(s) or Supplementary analyses when discussing socioeconomic inequities.

**Authors’ response:** We thank Reviewer 1 for the encouraging assessment and for the suggestions to improve clarity. We agree the distinction should be explicit. We edited the Figure 1 caption and text on p.5, lines 91–94.

2. Interpretation of concentration index results (Table 3): Table 3 appropriately summarises socioeconomic inequality using concentration indices. However, as a summary measure, the concentration index does not indicate how changes are distributed across individual wealth quintiles. Although supplementary analyses provide additional detail, briefly highlighting or cross-referencing these wealth-stratified patterns in the main text would improve clarity and accessibility for readers.

**Authors’ response:** We agree. In the Results, we now note this (on p.14, lines 280–282). In the Methods we also added a plain-language interpretation on p.19, lines 202–205.

3. Clarity of Statistical Interpretation: While the analytical approach (use of concentration indices and DID models) is methodologically appropriate, parts of the Results and Discussion sections are difficult to follow for non-specialist readers. Greater clarity in explaining (• the meaning of changes in concentration indices, • the interpretation of DID and DIDID estimates, and • their practical implications for equity) would improve the accessibility and impact of the paper.

**Authors’ response:** We have revised the manuscript for readability. In addition to the concentration-index interpretation above, we added a plain-language interpretation on p.11, lines 232–235. The Results and Discussion now state the direction of widening or narrowing gaps directly.

**Reviewer 2**

We thank the Reviewer for the detailed and rigorous methodological critique. We have taken the central recommendation seriously and, where a conventional causal difference-in-differences design and its diagnostics are not appropriate for our data, we have reframed the analysis as descriptive and associational and made the design’s inferential limits explicit, rather than presenting diagnostics that do not match the design. We respond below to the reviewer’s recommended revisions in turn (and, where relevant, to the related concerns raised earlier in the review).

1. Please clarify the informed consent procedures that were applied to the surveys.

**Authors’ response:** Addressed in the Methods ethics statement (p.7, lines 145–152).

2. Provide the full DID specification (equation, interaction terms, control variables, fixed effects); explain what makes it an “adaptation” and justify deviations from standard methods; specify whether two-way fixed effects are used and how standard errors are clustered.

**Authors’ response:** The full specification is presented as equation (1) in the Statistical Analysis, with every term and interaction defined in the text and derived in S3 Text in the supporting information document. We have clarified that standard errors were clustered at the cluster level and that the model includes survey-round (time) indicators and a district-level readiness indicator, but not district fixed effects. It is a pooled repeated cross-sectional interaction model rather than a two-way fixed effect DID. This is precisely what we mean by an “adaptation” of the DID approach, which we now state explicitly, together with the clarification that the design is not intended to evaluate a single policy with a discrete adoption date (p.11, lines 228–235; lines 241–245).

3. Clarify how high and low readiness districts are defined (threshold, aggregation method) and at what time point readiness is measured?

**Authors’ response:** Facility readiness is the district average of facility-level composite readiness scores (infrastructure, equipment, supplies, and commodities), computed separately for each linked survey round (2001/2000, 2010/2011, 2016/2017). For the descriptive analysis, the district distribution is divided into terciles (low/medium/high); for the multivariable models, we collapse this into low (bottom two terciles) and high (top tercile). This is now detailed in the Variables subsection.

4. At what point is readiness measured to classify districts, and does the classification remain fixed throughout 2001–2016 or change as readiness improves? How is time-varying treatment handled?

**Authors’ response:** The classification is time-varying. For each linked year, district-average readiness is computed from the corresponding facility survey, and districts are classified relative to that survey year’s distribution. Therefore, a district can move between readiness categories across waves. We now state this explicitly in the Variables subsection, and we note that this time-varying, contemporaneous nature is one reason the design departs from a standard DID with a fixed treatment group.

5. Describe whether you tested the parallel-trends assumption; can you provide event-study plots or pre-trend test results?

**Authors’ response:** These diagnostics are standard and highly informative when a treatment has a clearly timed adoption and observable pre-treatment periods. In our setting, readiness is measured contemporaneously at the district–year level from repeated facility surveys, with no common adoption date and with districts moving between categories over time; pre-treatment parallel-trends tests and event-study plots are therefore not directly applicable. Rather than report diagnostics that do not match the design, we have reframed the analysis as descriptive and added an explicit statement to the Limitations (*sixth* point).

6. Consider an anticipation test (lead terms or placebo tests with earlier ‘treatment’ dates).

**Authors’ response:** For the same reason (i.e., there is no discretely timed treatment), anticipation tests using lead terms and placebo treatment dates are not directly applicable to this design. We acknowledge this in the Limitations alongside the parallel trends point.

7. Did respondent characteristics (age, education, parity, urban–rural) change differentially between high- and low-readiness districts over 2001–2016? Consider transition-based estimates.

**Authors’ response:** We agree that this is an important feature of pooled repeated cross-sections. We now discuss it explicitly in the Limitations (*fifth* point): respondent composition shifted across rounds and may have shifted differentially across readiness groups; although these characteristics are adjusted for in the regression models, residual compositional differences may remain. Consistent with the descriptive scope of this revision, we did not estimate transition-based models, and we identify this as a direction for future work.

8. Clarify how the facility–household linkage was done, including the linkage rate and how distance was calculated.

**Authors’ response:** We have edited texts to clarify this (on p.9, lines 187–198; in supporting information under “Facility readiness: indicators, definitions, and district averages”). Because the household and facility surveys were sampled independently, households were not linked to individual or nearest facilities; instead, each district containing BMMS respondents was assigned the district-average readiness score from the same-round facility survey, representing the broader district service environment. All districts with respondents were successfully linked. The separate proximity measures (nearest public or private facility within one hour) are distinct from this readiness linkage and are based on respondents’ self-reported travel time to the nearest facility.

9. Consider sensitivity analysis with alternative readiness definitions, control groups, or subsamples to establish that results are not driven by arbitrary specification choices.

**Authors’ response:** We agree that sensitivity analyses strengthen a causal design. Given that we have reframed the study as descriptive and associational, we have not added new sensitivity analyses with alternative readiness thresholds, control groups, or subsamples in this revision. Instead, we have clarified the readiness definition, reduced causal language, and discussed explicitly the limitations of the chosen categorization and of the repeated cross-sectional design (*sixth* point in Limitations). We identify alternative-specification sensitivity analyses as a valuable next step for future research.

10. Consider accounting for staggered adoption if different districts experience improvements in readiness at different times.

**Authors’ response:** Staggered-adoption estimators presuppose a discrete treatment that different units adopt at different times. Because readiness here is a contemporaneous, time-varying district characteristic rather than an adopted intervention, staggered-adoption methods are not directly applicable. We note this explicitly in the Limitations.

11. Explain the extent of data missingness for key variables, whether it differs across groups or time, and how missing data were handled.

**Authors’ response:** We have added text to address this on p.11, lines 236–239.

12. If data are available, supplement structural readiness with process-quality or patient-experience indicators.

**Authors’ response:** Unfortunately, comparable process-quality and patient-experience indicators were not consistently available across the three linked survey rounds, so we were unable to incorporate them. We now state explicitly that our readiness measure captures structural capacity only and does not establish that structural readiness translated into the quality of care actually experienced by women (in Variables and Limitations subsections), and we flag this as an important direction for future research.

13. Consider stratified analyses (urban vs rural, administrative divisions, baseline poverty, public vs private dominance) to explore why high-readiness districts experienced faster increases in inequity.

**Authors’ response:** We appreciate this suggestion. Consistent with the descriptive scope of this revision, we did not add multiple new subgroup analyses; instead, we expanded the Discussion to consider plausible mechanisms and identified stratified analyses as a priority for future work.

14. In figures 2-3, add a legend explaining what the error bars represent (95% confidence interval or standard error?).

**Authors’ response:** We thank the reviewer for prompting this clarification. Fig 2 is an equiplot, in which each dot represents the level of service use within a wealth quintile and the horizontal distance between dots represents the socioeconomic gap; the markers are point estimates and do not include error bars or confidence intervals. We have added this clarification to the Fig 2 caption. For Fig 3 and 4, we have specified in the caption that the error bars represent 95% confidence intervals.

15. The discussion could be expanded to more thoroughly discuss mechanisms and compare findings with other low- and middle-income countries. Are the patterns unique to Bangladesh or generalizable?

**Authors’ response:** We have expanded the Discussion to situate the findings in the broader LMIC literature and to address generalizability. We now note that the pattern we observe raises the open question of whether it is unique to Bangladesh, and that the mechanisms we discuss (low absolute facility readiness, persistent demand-side and affordability barriers, and rapid private-sector growth) have been documented in other South Asian and sub-Saharan African settings, suggesting they may not be unique to Bangladesh.
