# Supplementary material for: Socioeconomic equity in maternal health services use in Bangladesh: The role of service readiness in health facilities during the period 2001–2016
Source: PLoS One. 2026 Jul 30;21(7):e0354897. doi: 10.1371/journal.pone.0354897 (PMC13422858; doi:10.1371/journal.pone.0354897)
Supplement: Peer review history

# Peer review: Decision Letter 0

**DOI:** 10.1371/journal.pone.0354897.r001

**Authors:** Abu Sayeed

15 Mar 2026

PONE-D-25-54631

Socioeconomic equity in maternal health services use in Bangladesh: The role of service readiness in health facilities during the period 2001–2016

PLOS One

Dear Dr. Ahsan,

Thank you for submitting your manuscript to PLOS ONE. After careful consideration, we feel that it has merit but does not fully meet PLOS ONE’s publication criteria as it currently stands. Therefore, we invite you to submit a revised version of the manuscript that addresses the points raised during the review process.

Please submit your revised manuscript by Apr 29 2026 11:59PM. If you will need more time than this to complete your revisions, please reply to this message or contact the journal office at plosone@plos.org. When you're ready to submit your revision, log on to https://www.editorialmanager.com/pone/ and select the 'Submissions Needing Revision' folder to locate your manuscript file.

Please include the following items when submitting your revised manuscript:

- A letter that responds to each point raised by the academic editor and reviewer(s). You should upload this letter as a separate file labeled 'Response to Reviewers'.
- A marked-up copy of your manuscript that highlights changes made to the original version. You should upload this as a separate file labeled 'Revised Manuscript with Track Changes'.
- An unmarked version of your revised paper without tracked changes. You should upload this as a separate file labeled 'Manuscript'.

If you would like to make changes to your financial disclosure, please include your updated statement in your cover letter. Guidelines for resubmitting your figure files are available below the reviewer comments at the end of this letter.

If applicable, we recommend that you deposit your laboratory protocols in protocols.io to enhance the reproducibility of your results. Protocols.io assigns your protocol its own identifier (DOI) so that it can be cited independently in the future. For instructions see: https://journals.plos.org/plosone/s/submission-guidelines#loc-laboratory-protocols. Additionally, PLOS ONE offers an option for publishing peer-reviewed Lab Protocol articles, which describe protocols hosted on protocols.io. Read more information on sharing protocols at https://plos.org/protocols?utm_medium=editorial-email&utm_source=authorletters&utm_campaign=protocols.

We look forward to receiving your revised manuscript.

Kind regards,

Abu Sayeed, MSc

Academic Editor

PLOS One

Journal Requirements:

When submitting your revision, we need you to address these additional requirements.

1. Please ensure that your manuscript meets PLOS ONE's style requirements, including those for file naming. The PLOS ONE style templates can be found at

https://journals.plos.org/plosone/s/file?id=wjVg/PLOSOne_formatting_sample_main_body.pdf and

https://journals.plos.org/plosone/s/file?id=ba62/PLOSOne_formatting_sample_title_authors_affiliations.pdf

2. Thank you for stating the following financial disclosure:

[This publication was produced with the support of the United States Agency for International Development (USAID) under the terms of USAID’s Data for Impact (D4I) associate award no. 7200AA18LA00008. We are grateful to the Carolina Population Center and MCH Department of the Gillings School of Global Public Health, whose funding and financial support have made this possible. Views expressed herein do not necessarily reflect the views of the U.S. Government or USAID.].

Please state what role the funders took in the study.  If the funders had no role, please state: ""The funders had no role in study design, data collection and analysis, decision to publish, or preparation of the manuscript.""

If this statement is not correct you must amend it as needed.

Please include this amended Role of Funder statement in your cover letter; we will change the online submission form on your behalf.

3. Please note that your Data Availability Statement is currently missing the DOI/accession number of each dataset OR a direct link to access each database. If your manuscript is accepted for publication, you will be asked to provide these details on a very short timeline. We therefore suggest that you provide this information now, though we will not hold up the peer review process if you are unable.

4. Please include your full ethics statement in the ‘Methods’ section of your manuscript file. In your statement, please include the full name of the IRB or ethics committee who approved or waived your study, as well as whether or not you obtained informed written or verbal consent. If consent was waived for your study, please include this information in your statement as well.

5. Please include captions for your Supporting Information files at the end of your manuscript, and update any in-text citations to match accordingly. Please see our Supporting Information guidelines for more information: http://journals.plos.org/plosone/s/supporting-information.

6. If the reviewer comments include a recommendation to cite specific previously published works, please review and evaluate these publications to determine whether they are relevant and should be cited. There is no requirement to cite these works unless the editor has indicated otherwise.

**Reviewers' comments:**

Reviewer's Responses to Questions

**Comments to the Author**

1. Is the manuscript technically sound, and do the data support the conclusions?

The manuscript must describe a technically sound piece of scientific research with data that supports the conclusions. Experiments must have been conducted rigorously, with appropriate controls, replication, and sample sizes. The conclusions must be drawn appropriately based on the data presented.

Reviewer #1: Yes

Reviewer #2: Yes

**********

2. Has the statistical analysis been performed appropriately and rigorously?

Reviewer #1: Yes

Reviewer #2: I Don't Know

**********

3. Have the authors made all data underlying the findings in their manuscript fully available?

The PLOS Data policy requires authors to make all data underlying the findings described in their manuscript fully available without restriction, with rare exception (please refer to the Data Availability Statement in the manuscript PDF file). The data should be provided as part of the manuscript or its supporting information, or deposited to a public repository. For example, in addition to summary statistics, the data points behind means, medians and variance measures should be available. If there are restrictions on publicly sharing data—e.g. participant privacy or use of data from a third party—those must be specified.

Reviewer #1: Yes

Reviewer #2: Yes

**********

4. Is the manuscript presented in an intelligible fashion and written in standard English?

PLOS ONE does not copyedit accepted manuscripts, so the language in submitted articles must be clear, correct, and unambiguous. Any typographical or grammatical errors should be corrected at revision, so please note any specific errors here.

Reviewer #1: Yes

Reviewer #2: Yes

**********

5. Review Comments to the Author

Please use the space provided to explain your answers to the questions above. You may also include additional comments for the author, including concerns about dual publication, research ethics, or publication ethics. (Please upload your review as an attachment if it exceeds 20,000 characters)

Reviewer #1: General Comments

This manuscript addresses an important and policy-relevant question regarding socioeconomic equity in maternal health service utilisation in Bangladesh and the role of facility readiness. The study uses nationally representative datasets and applies appropriate analytical methods. Overall, the analysis appears technically sound and the topic is highly relevant.

However, several aspects of the presentation and interpretation would benefit from clarification to improve accessibility for readers, particularly those without strong quantitative backgrounds. Strengthening the linkage between figures, tables, and interpretations, and improving clarity around equity measures, would enhance the manuscript’s contribution.

1. Attribution of equity trends to Figure 1

Figure 1 presents overall national trends in maternal health service utilisation across survey years but does not display wealth-stratified estimates. While wealth-quintile–specific patterns are presented elsewhere (e.g., Fig 2 and the Supplementary Information), statements referring to widening or narrowing gaps between the poorest and richest quintiles should not be attributed to Fig 1. The text would benefit from explicitly referencing the appropriate wealth-disaggregated figure(s) or Supplementary analyses when discussing socioeconomic inequities.

2. Interpretation of concentration index results (Table 3)

Table 3 appropriately summarises socioeconomic inequality using concentration indices. However, as a summary measure, the concentration index does not indicate how changes are distributed across individual wealth quintiles. Although supplementary analyses provide additional detail, briefly highlighting or cross-referencing these wealth-stratified patterns in the main text would improve clarity and accessibility for readers.

3. Clarity of Statistical Interpretation

While the analytical approach (use of concentration indices and DID models) is methodologically appropriate, parts of the Results and Discussion sections are difficult to follow for non-specialist readers.

Greater clarity in explaining:

• the meaning of changes in concentration indices,

• the interpretation of DID and DIDID estimates, and

• their practical implications for equity,

would improve the accessibility and impact of the paper

Reviewer #2: The study tackles an important research question and uses valuable longitudinal data. The study aligns with the Sustainable Development Goal push for universal health coverage by 2030 and provides useful evidence for Bangladesh and other low- and middle -income countries. The unexpected association between higher facility readiness and widening socioeconomic gaps in facility delivery is policy-relevant and challenges the implicit assumption that supply-side improvements inherently reduce inequity. If valid, this finding has significant implications for universal health coverage strategies. middle-income countries.

The manuscript is generally well-written and organized, with clear tables and figures.

However, there are major problems with the methods that weaken the strength of its causal claims regarding the effect of facility readiness on equity as follows:

1. The adapted DID approach is not clearly explained, lacks proper diagnostic checks, and does not justify why it differs from standard methods. You describe having used "an adaptation of the Difference-in-Differences (DID) model" but provide insufficient detail about what constitutes this "adaptation" and why it deviates from standard DID.

It would have been clearer to specify whether two-way fixed effects are used and how standard errors are clustered

2. Essential methodological details are either missing or insufficiently explained, particularly concerning the DID specification, treatment definition, and assumption testing.

3.The statistical methods section requires substantial expansion to facilitate replication and enable assessment of validity. The paper does not report standard regression diagnostics (coefficients, standard errors, sample sizes, and goodness-of-fit statistics for all models.). The paper references appendices (e.g., "Appendix 4") that contain full regression tables, but these are not included in the version reviewed.

3. Does a district's readiness classification remain fixed across the study period, or does it change? If readiness improves in a "low readiness" district, does it remain in the low group or switch to the high group?

If readiness changes over time, how is this time-varying treatment handled?

4. The paper does not discuss what time-varying confounders might threaten validity or how they are addressed. For example, what other maternal health policies or programs were implemented in Bangladesh during 2001–2016? How might these confound the readiness-equity relationship?

5.The study uses pooled cross-sectional data from three survey rounds (2001, 2010, 2016), which may introduce changes in sample composition across waves. Did you assess for compositional stability?

6.The paper states that household surveys are "linked to corresponding health facility surveys" but does not clearly describe the linkage method. Are households linked to the nearest facility or to facilities within a certain distance? How is distance calculated (straight-line or travel time?). What proportion of households could be successfully linked?

The linkage method affects the validity of the readiness-equity relationship.

7. The paper does not report on the magnitude of missing data and how they were handled.

8.The study assumes that structural readiness ((equipment, staff, medicines) translates to quality along with utilization, but this link is not empirically established in this paper.

On ethical aspects, the paper notes "No competing interests reported," but does not discuss informed consent procedures for the surveys.

I recommend the following major revisions as necessary to either, strengthen the quasi-experimental design with proper diagnostics and sensitivity analyses, or to reframe the analysis as descriptive rather than causal:

Firstly, please clarify the informed consent procedures that were applied to the surveys.

1.Provide the full DID specification including the exact regression equation used for the DID analysis, all interaction terms, control variables, and fixed effects. Explain what makes the specification an "adaptation" and justify any variations from standard methods.

2.Clarify how "high" and "low" readiness districts are defined (threshold, aggregation method). At what time point readiness is measured?

3. Clarify the treatment timing: At what point in time is facility readiness measured to classify districts as "high" or "low"? Does this classification remain fixed throughout 2001–2016, or does it change as readiness improves?

4. Describe whether you tested the parallel trends assumption. Can you provide event-study plots or pre-trend test results?

5.Consider anticipation test: Is there evidence that districts or individuals anticipated readiness improvements and changed behaviour before readiness actually improved? Standard practice is to include lead terms in the regression or conduct placebo tests with earlier "treatment" dates.

6 Assess compositional stability: Did the characteristics of survey respondents (age, education, parity, urban-rural) change differentially between high and low readiness districts during 2001–2016? Report whether the characteristics of respondents (age, education, parity, urban-rural residence) changed differentially between high and low readiness districts over time. Consider transition-based estimates and if compositional changes are considerable.

7. Clarify how facility-household linkage was done, including details of linkage rate and how distance was calculated.

8. Consider doing sensitivity analysis with alternative readiness definitions, control groups or subsamples. This is important for establishing that results are not driven by arbitrary specification choices

9. Consider accounting of staggered adoption in the analysis in case different districts experience improvements in readiness at different times.

10. Explain the extent of data missingness for key variables, whether it differs across groups or time period and how missing data were handled (complete case analysis? imputation?)

11.If data are available, supplement structural readiness measures with process quality or patient experience indicators to better understand the relationship between readiness and equity.

12. To enhance the paper, consider doing stratified analyses to explore why high-readiness districts experienced faster increases in inequity. Examine whether the readiness-equity relationship differs by: Urban vs. rural settings, administrative divisions or regions, baseline poverty levels or Public vs. private facility dominance.

13. In figures 2-3, add a legend explaining what the error bars represent (95% Confidence interval or Standard error?)

Finally, the discussion could be expanded to more thoroughly discuss mechanisms and compare findings with other Low- and middle-income countries. Are the patterns unique to Bangladesh or generalizable?

Congratulations and I wish you success in your publication journey.

**********

6. PLOS authors have the option to publish the peer review history of their article (what does this mean?). If published, this will include your full peer review and any attached files.

If you choose “no”, your identity will remain anonymous but your review may still be made public.

**Do you want your identity to be public for this peer review?**  For information about this choice, including consent withdrawal, please see our Privacy Policy.

Reviewer #1: No

Reviewer #2: No

**********

[NOTE: If reviewer comments were submitted as an attachment file, they will be attached to this email and accessible via the submission site. Please log into your account, locate the manuscript record, and check for the action link "View Attachments". If this link does not appear, there are no attachment files.]

To ensure your figures meet our technical requirements, please review our figure guidelines: https://journals.plos.org/plosone/s/figures

You may also use PLOS’s free figure tool, NAAS, to help you prepare publication quality figures: https://journals.plos.org/plosone/s/figures#loc-tools-for-figure-preparation.

NAAS will assess whether your figures meet our technical requirements by comparing each figure against our figure specifications.

# Author comment: Author response to Decision Letter 1

**DOI:** 10.1371/journal.pone.0354897.r002

22 Jun 2026

Authors’’ response to Editorial and Reviewers’ comments

Manuscript ID: PONE-D-25-54631

Editorial Office

1. Please ensure that your manuscript meets PLOS ONE's style requirements, including those for file naming. The PLOS ONE style templates can be found at

https://journals.plos.org/plosone/s/file?id=wjVg/PLOSOne_formatting_sample_main_body.pdf and https://journals.plos.org/plosone/s/file?id=ba62/PLOSOne_formatting_sample_title_authors_affiliations.pdf.

Authors’ response: We have formatted the manuscript, tables, figures, and file names in line with the PLOS ONE style templates for the main body and for the title/authors/affiliations, and have ensured the submission meets PLOS ONE's style and file-naming requirements.

2. Thank you for stating the following financial disclosure: [This publication was produced with the support of the United States Agency for International Development (USAID) under the terms of USAID’s Data for Impact (D4I) associate award no. 7200AA18LA00008. We are grateful to the Carolina Population Center and MCH Department of the Gillings School of Global Public Health, whose funding and financial support have made this possible. Views expressed herein do not necessarily reflect the views of the U.S. Government or USAID.]. Please state what role the funders took in the study. If the funders had no role, please state: ""The funders had no role in study design, data collection and analysis, decision to publish, or preparation of the manuscript."" If this statement is not correct you must amend it as needed. Please include this amended Role of Funder statement in your cover letter; we will change the online submission form on your behalf.

Authors’ response: We added “The funders had no role in study design, data collection and analysis, decision to publish, or preparation of the manuscript.” in the financial disclosure.

3. Please note that your Data Availability Statement is currently missing the DOI/accession number of each dataset OR a direct link to access each database. If your manuscript is accepted for publication, you will be asked to provide these details on a very short timeline. We therefore suggest that you provide this information now, though we will not hold up the peer review process if you are unable.

Authors’ response: Data Availability Statement updated by providing the required information.

4. Please include your full ethics statement in the ‘Methods’ section of your manuscript file. In your statement, please include the full name of the IRB or ethics committee who approved or waived your study, as well as whether or not you obtained informed written or verbal consent. If consent was waived for your study, please include this information in your statement as well.

Authors’ response: Included on p.7, lines 144–151.

5. Please include captions for your Supporting Information files at the end of your manuscript, and update any in-text citations to match accordingly. Please see our Supporting Information guidelines for more information: http://journals.plos.org/plosone/s/supporting-information.

Authors’ response: We have added a "Supporting Information" section at the end of the manuscript (pp.34–35, lines 832–840).

6. If the reviewer comments include a recommendation to cite specific previously published works, please review and evaluate these publications to determine whether they are relevant and should be cited. There is no requirement to cite these works unless the editor has indicated otherwise.

Authors’ response: Not applicable.

Reviewer 1

1. Figure 1 presents overall national trends in maternal health service utilisation across survey years but does not display wealth-stratified estimates. While wealth-quintile–specific patterns are presented elsewhere (e.g., Fig 2 and the Supplementary Information), statements referring to widening or narrowing gaps between the poorest and richest quintiles should not be attributed to Fig 1. The text would benefit from explicitly referencing the appropriate wealth-disaggregated figure(s) or Supplementary analyses when discussing socioeconomic inequities.

Authors’ response: We thank Reviewer 1 for the encouraging assessment and for the suggestions to improve clarity. We agree the distinction should be explicit. We edited the Figure 1 caption and text on p.5, lines 91–94.

2. Interpretation of concentration index results (Table 3): Table 3 appropriately summarises socioeconomic inequality using concentration indices. However, as a summary measure, the concentration index does not indicate how changes are distributed across individual wealth quintiles. Although supplementary analyses provide additional detail, briefly highlighting or cross-referencing these wealth-stratified patterns in the main text would improve clarity and accessibility for readers.

Authors’ response: We agree. In the Results, we now note this (on p.14, lines 280–282). In the Methods we also added a plain-language interpretation on p.19, lines 202–205.

3. Clarity of Statistical Interpretation: While the analytical approach (use of concentration indices and DID models) is methodologically appropriate, parts of the Results and Discussion sections are difficult to follow for non-specialist readers. Greater clarity in explaining (• the meaning of changes in concentration indices, • the interpretation of DID and DIDID estimates, and • their practical implications for equity) would improve the accessibility and impact of the paper.

Authors’ response: We have revised the manuscript for readability. In addition to the concentration-index interpretation above, we added a plain-language interpretation on p.11, lines 232–235. The Results and Discussion now state the direction of widening or narrowing gaps directly.

Reviewer 2

We thank the Reviewer for the detailed and rigorous methodological critique. We have taken the central recommendation seriously and, where a conventional causal difference-in-differences design and its diagnostics are not appropriate for our data, we have reframed the analysis as descriptive and associational and made the design’s inferential limits explicit, rather than presenting diagnostics that do not match the design. We respond below to the reviewer’s recommended revisions in turn (and, where relevant, to the related concerns raised earlier in the review).

1. Please clarify the informed consent procedures that were applied to the surveys.

Authors’ response: Addressed in the Methods ethics statement (p.7, lines 145–152).

2. Provide the full DID specification (equation, interaction terms, control variables, fixed effects); explain what makes it an “adaptation” and justify deviations from standard methods; specify whether two-way fixed effects are used and how standard errors are clustered.

Authors’ response: The full specification is presented as equation (1) in the Statistical Analysis, with every term and interaction defined in the text and derived in S3 Text in the supporting information document. We have clarified that standard errors were clustered at the cluster level and that the model includes survey-round (time) indicators and a district-level readiness indicator, but not district fixed effects. It is a pooled repeated cross-sectional interaction model rather than a two-way fixed effect DID. This is precisely what we mean by an “adaptation” of the DID approach, which we now state explicitly, together with the clarification that the design is not intended to evaluate a single policy with a discrete adoption date (p.11, lines 228–235; lines 241–245).

3. Clarify how high and low readiness districts are defined (threshold, aggregation method) and at what time point readiness is measured?

Authors’ response: Facility readiness is the district average of facility-level composite readiness scores (infrastructure, equipment, supplies, and commodities), computed separately for each linked survey round (2001/2000, 2010/2011, 2016/2017). For the descriptive analysis, the district distribution is divided into terciles (low/medium/high); for the multivariable models, we collapse this into low (bottom two terciles) and high (top tercile). This is now detailed in the Variables subsection.

4. At what point is readiness measured to classify districts, and does the classification remain fixed throughout 2001–2016 or change as readiness improves? How is time-varying treatment handled?

Authors’ response: The classification is time-varying. For each linked year, district-average readiness is computed from the corresponding facility survey, and districts are classified relative to that survey year’s distribution. Therefore, a district can move between readiness categories across waves. We now state this explicitly in the Variables subsection, and we note that this time-varying, contemporaneous nature is one reason the design departs from a standard DID with a fixed treatment group.

5. Describe whether you tested the parallel-trends assumption; can you provide event-study plots or pre-trend test results?

Authors’ response: These diagnostics are standard and highly informative when a treatment has a clearly timed adoption and observable pre-treatment periods. In our setting, readiness is measured contemporaneously at the district–year level from repeated facility surveys, with no common adoption date and with districts moving between categories over time; pre-treatment parallel-trends tests and event-study plots are therefore not directly applicable. Rather than report diagnostics that do not match the design, we have reframed the analysis as descriptive and added an explicit statement to the Limitations (sixth point).

6. Consider an anticipation test (lead terms or placebo tests with earlier ‘treatment’ dates).

Authors’ response: For the same reason (i.e., there is no discretely timed treatment), anticipation tests using lead terms and placebo treatment dates are not directly applicable to this design. We acknowledge this in the Limitations alongside the parallel trends point.

7. Did respondent characteristics (age, education, parity, urban–rural) change differentially between high- and low-readiness districts over 2001–2016? Consider transition-based estimates.

Authors’ response: We agree that this is an important feature of pooled repeated cross-sections. We now discuss it explicitly in the Limitations (fifth point): respondent composition shifted across rounds and may have shifted differentially across readiness groups; although these characteristics are adjusted for in the regression models, residual compositional differences may remain. Consistent with the descriptive scope of this revision, we did not estimate transition-based models, and we identify this as a direction for future work.

8. Clarify how the facility–household linkage was done, including the linkage rate and how distance was calculated.

Authors’ response: We have edited texts to clarify this (on p.9, lines 187–198; in supporting information under “Facility readiness: indicators, definitions, and district averages”). Because the household and facility surveys were sampled independently, households were not linked to individual or nearest facilities; instead, each district containing BMMS respondents was assigned the district-average readiness score from the same-round facility survey, representing the broader district service environment. All districts with respondents were successfully linked. The separate proximity measures (nearest public or private facility within one hour) are distinct from this readiness linkage and are based on respondents’ self-reported travel time to the nearest facility.

9. Consider sensitivity analysis with alternative readiness definitions, control groups, or subsamples to establish that results are not driven by arbitrary specification choices.

Authors’ response: We agree that sensitivity analyses strengthen a causal design. Given that we have reframed the study as descriptive and associational, we have not added new sensitivity analyses with alternative readiness thresholds, control groups, or subsamples in this revision. Instead, we have clarified the readiness definition, reduced causal language, and discussed explicitly the limitations of the chosen categorization and of the repeated cross-sectional design (sixth point in Limitations). We identify alternative-specification sensitivity analyses as a valuable next step for future research.

10. Consider accounting for staggered adoption if different districts experience improvements in readiness at different times.

Authors’ response: Staggered-adoption estimators presuppose a discrete treatment that different units adopt at different times. Because readiness here is a contemporaneous, time-varying district characteristic rather than an adopted intervention, staggered-adoption methods are not directly applicable. We note this explicitly in the Limitations.

11. Explain the extent of data missingness for key variables, whether it differs across groups or time, and how missing data were handled.

Authors’ response: We have added text to address this on p.11, lines 236–239.

12. If data are available, supplement structural readiness with process-quality or patient-experience indicators.

Authors’ response: Unfortunately, comparable process-quality and patient-experience indicators were not consistently available across the three linked survey rounds, so we were unable to incorporate them. We now state explicitly that our readiness measure captures structural capacity only and does not establish that structural readiness translated into the quality of care actually experienced by women (in Variables and Limitations subsections), and we flag this as an important direction for future research.

13. Consider stratified analyses (urban vs rural, administrative divisions, baseline poverty, public vs private dominance) to explore why high-readiness districts experienced faster increases in inequity.

Authors’ response: We appreciate this suggestion. Consistent with the descriptive scope of this revision, we did not add multiple new subgroup analyses; instead, we expanded the Discussion to consider plausible mechanisms and identified stratified analyses as a priority for future work.

14. In figures 2-3, add a legend explaining what the error bars represent (95% confidence interval or standard error?).

Authors’ response: We thank the reviewer for prompting this clarification. Fig 2 is an equiplot, in which each dot represents the level of service use within a wealth quintile and the horizontal distance between dots represents the socioeconomic gap; the markers are point estimates and do not include error bars or confidence intervals. We have added this clarification to the Fig 2 caption. For Fig 3 and 4, we have specified in the caption that the error bars represent 95% confidence intervals.

15. The discussion could be expanded to more thoroughly discuss mechanisms and compare findings with other low- and middle-income countries. Are the patterns unique to Bangladesh or generalizable?

Authors’ response: We have expanded the Discussion to situate the findings in the broader LMIC literature and to address generalizability. We now note that the pattern we observe raises the open question of whether it is unique to Bangladesh, and that the mechanisms we discuss (low absolute facility readiness, persistent demand-side and affordability barriers, and rapid private-sector growth) have been documented in other South Asian and sub-Saharan African settings, suggesting they may not be unique to Bangladesh.

Attachment Submitted filename: Response_to_Reviewers_R1.docx

# Editor report: Decision Letter 1

**DOI:** 10.1371/journal.pone.0354897.r003

**Authors:** Abu Sayeed

15 Jul 2026

Socioeconomic equity in maternal health services use in Bangladesh: The role of service readiness in health facilities during the period 2001–2016

PONE-D-25-54631R1

Dear Dr. Karar Zunaid Ahsan,

We’re pleased to inform you that your manuscript has been judged scientifically suitable for publication and will be formally accepted for publication once it meets all outstanding technical requirements.

Within one week, you’ll receive an e-mail detailing the required amendments. When these have been addressed, you’ll receive a formal acceptance letter and your manuscript will be scheduled for publication.

An invoice will be generated when your article is formally accepted. Please note, if your institution has a publishing partnership with PLOS and your article meets the relevant criteria, all or part of your publication costs will be covered. Please make sure your user information is up-to-date by logging into Editorial Manager at Editorial Manager® and clicking the ‘Update My Information' link at the top of the page. For questions related to billing, please contact billing support.

If your institution or institutions have a press office, please notify them about your upcoming paper to help maximize its impact. If they’ll be preparing press materials, please inform our press team as soon as possible -- no later than 48 hours after receiving the formal acceptance. Your manuscript will remain under strict press embargo until 2 pm Eastern Time on the date of publication. For more information, please contact onepress@plos.org.

Kind regards,

Abu Sayeed, MSc

Academic Editor

PLOS One

Additional Editor Comments:

Thank you for your careful revision of the manuscript. You have addressed all of the reviewers' comments thoroughly and appropriately, and the manuscript has improved substantially. I have no further comments and believe that the manuscript is now suitable for publication in its current form.

Reviewers' comments:

# Editor report: Acceptance letter

**DOI:** 10.1371/journal.pone.0354897.r004

**Authors:** Abu Sayeed

PONE-D-25-54631R1

PLOS One

Dear Dr. Ahsan,

I'm pleased to inform you that your manuscript has been deemed suitable for publication in PLOS One. Congratulations! Your manuscript is now being handed over to our production team.

At this stage, our production department will prepare your paper for publication. This includes ensuring the following:

* All references, tables, and figures are properly cited

* All relevant supporting information is included in the manuscript submission,

* There are no issues that prevent the paper from being properly typeset

You will receive further instructions from the production team, including instructions on how to review your proof when it is ready. Please keep in mind that we are working through a large volume of accepted articles, so please give us a few days to review your paper and let you know the next and final steps.

Lastly, if your institution or institutions have a press office, please let them know about your upcoming paper now to help maximize its impact. If they'll be preparing press materials, please inform our press team within the next 48 hours. Your manuscript will remain under strict press embargo until 2 pm Eastern Time on the date of publication. For more information, please contact onepress@plos.org.

You will receive an invoice from PLOS for your publication fee after your manuscript has reached the completed accept phase. If you receive an email requesting payment before acceptance or for any other service, this may be a phishing scheme. Learn how to identify phishing emails and protect your accounts at https://explore.plos.org/phishing.

If we can help with anything else, please email us at customercare@plos.org.

Thank you for submitting your work to PLOS ONE and supporting open access.

Kind regards,

PLOS ONE Editorial Office Staff

on behalf of

Mr. Abu Sayeed

Academic Editor

PLOS One
